# Augmenting tomato functional genomics with a genome-wide induced genetic variation resource

**DOI:** 10.3389/fpls.2023.1290937

**Published:** 2024-01-24

**Authors:** Prateek Gupta, Pankaj Singh Dholaniya, Kunnappady Princy, Athira Sethu Madhavan, Yellamaraju Sreelakshmi, Rameshwar Sharma

**Affiliations:** ^1^ Repository of Tomato Genomics Resources, Department of Plant Sciences, University of Hyderabad, Hyderabad, India; ^2^ Department of Biological Sciences, SRM University-AP, Amaravati, Andhra Pradesh, India; ^3^ Department of Biotechnology and Bioinformatics, University of Hyderabad, Hyderabad, India

**Keywords:** codon usage, EMS, functional genomics, genetic code, mutagenesis, *Solanum lycopersicum*, tomato, WGS

## Abstract

Induced mutations accelerate crop improvement by providing novel disease resistance and yield alleles. However, the alleles with no perceptible phenotype but have an altered function remain hidden in mutagenized plants. The whole-genome sequencing (WGS) of mutagenized individuals uncovers the complete spectrum of mutations in the genome. Genome-wide induced mutation resources can improve the targeted breeding of tomatoes and facilitate functional genomics. In this study, we sequenced 132 doubly ethyl methanesulfonate (EMS)-mutagenized lines of tomato and detected approximately 41 million novel mutations and 5.5 million short InDels not present in the parental cultivar. Approximately 97% of the genome had mutations, including the genes, promoters, UTRs, and introns. More than one-third of genes in the mutagenized population had one or more deleterious mutations predicted by Sorting Intolerant From Tolerant (SIFT). Nearly one-fourth of deleterious genes mapped on tomato metabolic pathways modulate multiple pathway steps. In addition to the reported GC>AT transition bias for EMS, our population also had a substantial number of AT>GC transitions. Comparing mutation frequency among synonymous codons revealed that the most preferred codon is the least mutagenic toward EMS. The validation of a potato leaf-like mutation, reduction in carotenoids in ζ-carotene isomerase mutant fruits, and chloroplast relocation loss in phototropin1 mutant validated the mutation discovery pipeline. Our database makes a large repertoire of mutations accessible to functional genomics studies and breeding of tomatoes.

## Introduction

The impending climate changes and burgeoning human population have placed a marked emphasis on doubling global food production by 2050. Parallel to cereals, which provide the most calorific values, there is an impetus to increase the yield and nutraceuticals in vegetable crops. Tomato, an important globally grown crop, is enriched in several nutraceuticals that are lacking in cereals. Like other crops, in tomatoes, the domestication pressure led to the loss in nutritional value, flavor, and disease resistance due to genetic erosion (Tanksley and McCouch, 1997; Bauchet and Causse, 2012; Tieman et al., 2017). The genome resequencing of a large number of tomato cultivars, along with several wild relatives, has highlighted the extent of loss of genetic diversity in modern tomato cultivars during domestication (Aflitos et al., 2014; Lin et al., 2014; Gupta et al., 2020a).

The genetic diversity of a domesticated crop can be augmented by the introgression of chromosomal segments from wild relatives or by *de novo* induction of diversity by induced mutagenesis (Kulus, 2018). Before the advent of the genomics era, the diversity induced by mutagenesis was harnessed by the visual selection of the mutants displaying desired traits and backcrossing to the parent. The availability of genome sequences of crop plants expanded the scope of introgression of induced mutations by providing molecular markers (Foolad, 2007; Simko et al., 2021). It also facilitated functional genomic analysis of genes mutated by chemical/radiation mutagens, t-DNA, or transposon insertion.

Compared to induced mutagenesis, the t-DNA or transposon-mediated disruption of gene function remained limited to species easily amenable to the transformation, such as rice and *Arabidopsis* (Provart et al., 2016; Ram et al., 2019). In contrast, induced mutagenesis is not species-specific, but being random, it creates a large number of mutations across the whole genome (Leitao, 2011). The development of reverse genetic tools, particularly Targeting Induced Local Lesions in Genomes (TILLING) based on PCR screening, rendered it possible to identify the mutation in any gene (McCallum et al., 2000). TILLING was not limited to detecting induced mutations; it was also applied to detect the single-nucleotide polymorphisms (SNPs) present in natural accessions (Comai et al., 2004). The fast detection of mutations in any gene immensely widened the scope of induced mutagenesis in crop plants.

To exploit the potential of TILLING, mutant populations were generated in several crop plants, such as rice, wheat, tomato, and maize (Gupta et al., 2018; Jacob et al., 2018) (**Supplementary Table 1**). In tomatoes, ethyl methanesulfonate (EMS)-mutagenized populations for TILLING were made for several cultivars such as M82 (Menda et al., 2004; Piron et al., 2010), Red Setter (Minoia et al., 2010), TPAADASU (Gady et al., 2009), Micro-Tom (Okabe et al., 2011), and Arka Vikas (Sharma et al., 2021). An advantage of TILLING was that the pooled genomic DNA could be scanned for mutations in the target gene. Notwithstanding the convenience, TILLING is laborious and slow, as scanning of mutation at best could be performed for 1.0–1.5 kb genomic DNA, with no possibility of multiplexing. In addition, the precise identification of the mutated base needed Sanger sequencing.

The above drawback was obviated by next-generation sequencing (NGS), where the mutagenized population could be analyzed using “TILLING by sequencing”. Herein, the multi-dimensionally pooled genomic DNA was subjected to PCR to amplify a given gene. The PCR products were then pooled, followed by sequencing using NGS. The output data were analyzed to reveal rare induced mutations. The NGS-based TILLING was used for rice and wheat (Tsai et al., 2011), tomato (Rigola et al., 2009; Gupta et al., 2017), poplar (Marroni et al., 2011), peanut (Guo et al., 2015), and soybean (Tsuda et al., 2015). Though NGS accelerated the identification of mutations compared to conventional TILLING, the scope of NGS-based TILLING remained limited. The identification of a heterozygous mutation was limited to 64X pooled DNA. Most software was not robust enough to identify the mutations from the background noise, barring a few such as CAMBA and GATK (Gupta et al., 2017).

The main drawback of TILLING was that mutations in only a few genes could be analyzed in one cycle, though the mutant lines bore mutations across the genome. The best approach is to identify the mutations across the genome by resequencing the genome. The reduction in whole-genome sequencing (WGS) cost made this approach feasible. However, the very large genomes of crop plants such as maize and higher ploidy levels of wheat made WGS an expensive approach. Presuming that most mutations in intergenic regions and introns do not elicit a phenotype, the sequencing was selectively performed for the exome for maize and wheat. The whole-exome sequencing (WES) efficiently identified mutations in rice (a small genome plant) and wheat (a large genome plant) (Henry et al., 2014). In tetraploid and hexaploid wheat cultivars, the WES of 2,735 mutagenized lines identified more than 10 million mutations (Krasileva et al., 2017). The WES of 2,090 mutant lines of KN9204 wheat identified 1,383 EMS-type SNPs per line (Wang et al., 2023). Similarly, the WES of pollen-mutagenized maize plants identified nearly 0.2 million mutations in 1,086 M_1_ lines (Lu et al., 2018).

Though the WES is more cost-effective than WGS, it has an inherent cost of capture probe designing and determining its efficiency. In maize, the efficiency of exome capture probes was approximately 83% (Lu et al., 2018). In addition, WES omits the significant number of mutations present in the intergenic regions, promoters, and introns (Belkadi et al., 2015). In tomato ‘Micro-Tom’ using exome capture, 241,391 mutations were identified in 95 M_2_ lines (Yano et al., 2019). While the mutations in the genic region are the ones that affect the traits, emerging evidence has indicated that mutations in introns, promoters, and intergenic regions can also influence the trait. In tomatoes, a point mutation in the promoter of the *1-aminocyclopropane carboxylase2* gene reduced ethylene emission from the fruits and considerably prolonged the shelf life (Sharma et al., 2021). Thus, the potential of a mutagenized population can be best unlocked by the WGS, as it provides a repertoire of mutations across the genome.

In this study, we present the WGS of 132 EMS-mutagenized lines of tomato. Our analysis reveals that in addition to GC>AT transitions, mutagenesis also caused a substantial number of AT>GC transitions. We gene-indexed 41 million novel mutations and 5.5 million InDels that were not present in the parental cultivar in an open-access database called Induced Tomato Genomic Variations (ITGV; http://psd.uohyd.ac.in/itgv/). The ITGV allows users to search for mutations in the desired gene and visualize the mutation’s nature, functional effects, and protein alignment. The above collection of mutant lines provides the scientific community with a genome-wide resource for tomato mutations. The mutant collection can be used for functional genomics of tomatoes and by breeders for trait improvement.

## Materials and methods

### Mutant population and DNA isolation

The doubly mutagenized (120 mM EMS) tomato (cultivar Arka Vikas) lines used in this study were the M_4_ progeny of the M_2_M_2_ population described earlier by Gupta et al. (2017). Briefly, the seeds from 1,000 M_2_ lines were remutagenized with 120 mm EMS, and M_2_M_1_ plants were grown in an open field. The M_2_M_2_ seeds were harvested and were sown to raise the M_2_M_2_ plants. The M_2_M_2_ generation was carried forward to M_2_M_4_ plants. The juvenile leaves from the individual M_2_M_2_ plants were collected for genomic DNA isolation. Leaf samples for genomic DNA were collected from 132 randomly selected M_2_M_4_ plants. Genomic DNA was isolated using DNeasy Plant Mini Kit (Qiagen, Hilden, Germany) and in-house lab protocol (Sreelakshmi et al., 2010; Gupta et al., 2020b) (**Supplementary Figure 1**).

### DNA sequencing, read mapping, and variant calling

Whole-genome sequencing was performed on the HiSeqX sequencing system (Illumina) by GeneWiz Inc. (South Plainfield, NJ, USA) following the manufacturer’s protocol. For each mutant line, a minimum of ~200 million reads (30 Gb/sample) with a target of a minimum of 25 Gb > Q30 were generated. The raw reads were filtered using fastp software (v0.19.5) using parameters -M 30 -3 -5. The 2X 150-bp reads were mapped on *Solanum lycopersicum* cv. Heinz version SL3.0 using BWA-MEM (0.7.17) (Li and Durbin, 2009). The data analysis and variant calling were performed as described by Gupta et al. (2020a).

Briefly, GATK (4.0.3.0) was used for generating BAM files, PCR duplicate removal, and variant calling. Variant filtration was carried out using the GATK VariantFiltration command. The parameters used for the filtering for SNPs were QualByDepth (QD < 2), FisherStrand (FS > 60), RMSMappingQuality (MQ < 40), MQRankSum (−12.5), and ReadPosRankSum (−8.0); those for InDels were QualByDepth (QD < 2), FisherStrand (FS > 200), and ReadPosRankSum (−20.0) (https://gatk.broadinstitute.org/hc/en-us/articles/360035531112?id=6925). The SNPs present in the parental cultivar Arka Vikas were subtracted from all 132 mutant lines. The resulting vcf (variant calling format) files were annotated using the SIFT4G algorithm (Vaser et al., 2016; **Supplementary Figure 2**).

The effect of base substitutions on protein function was determined by SIFT4G (SIFT score ≤0.05 is considered deleterious) using the SIFT4G-ITAG3.2 genome reference database generated by Gupta et al. (2020a). The deleteriously predicted genes by SIFT4G (≤0.05) were mapped on the tomato metabolic pathway (ITAG annotation 3.2; PlantCYC database 5.0.1) (Schläpfer et al., 2017). The genome-wide distribution of SNPs was generated using CircosVCF (Drori et al., 2017). Tomato microRNA locus coordinates were from the PmiREN database (https://www.pmiren.com/) (Guo et al., 2020). The circular plot of amino acid was generated using the chorddiag package in R (https://github.com/mattflor/chorddiag).

### Carotenoid analysis and chloroplast movement

Carotenoid extraction and analysis were carried out as described by Gupta et al. (2015). Briefly, freeze-dried fruit tissue (~150 mg) was homogenized; to the homogenate, 1.5 mL of chloroform:dichloromethane (2:1, v/v) was added. The resultant suspension was mixed for 20 min using a thermomixer at 1,000 rpm at 4°C. After that, for phase separation, 0.5 mL of 1 M sodium chloride solution was added, and the contents were mixed by inversion. After centrifugation at 5,000 *g* for 10 min, the organic phase was collected. The aqueous phase was re-extracted with 0.75 mL of chloroform:dichloromethane (2:1, v/v) and centrifuged, and again, the organic phase was collected. Both organic phases were pooled, dried by centrifugal evaporation, and re-dissolved in 1 mL of methanol/MTBE (25/75, v/v). From this, a 20-μL aliquot was used for injection into high-performance liquid chromatography (HPLC). Carotenoids were analyzed by reversed-phase HPLC using a C30 column.

Chloroplast movement in tomato leaves was monitored by measuring the red light transmittance through leaf discs using a microplate reader (Biotek, Synergy HT, Hampton, NH, USA) at 25°C as described by Kilambi et al. (2021). The excised tomato leaf discs of phototropin mutants and Arka Vikas parental plants were placed on 0.5% agar (w/v) in 96-well microplates for 12 hours in the dark. After that, the red light transmittance was continually recorded in a microplate reader for 30 min. The leaf discs were then irradiated with weak blue light (3.2 μmol m^−2^ s^−1^) for 80 min to elicit chloroplast accumulation, and the shift in red light transmittance was recorded. Next, the leaf discs were exposed to intense blue light (80 μmol m^−2^ s^−1^) to induce chloroplast avoidance, and the shift in red light transmittance was recorded for the next 80 min. The chloroplast accumulation and avoidance data for phototropin1 mutants and *Nps1* mutants were expressed as percent chloroplast movement relative to the Arka Vikas parental control.

### ITGV database

The ITGV database was made as described previously by Gupta et al. (2020a). Briefly, the database runs on the XAMPP Apache server. The database was developed using MySQL/MariaDB relational database management system, HTML, CSS, PHP, and JavaScript libraries. Data search and submission queries were built using SQL. The genome browser “JBrowse” was also integrated into the ITGV database to visualize the variants. The VCF files for all the accessions can be downloaded from the download data option in the database. Interested researchers can search ITGV online to identify mutations and SIFT scores in their target genes. The mutant line seeds can be requested using the MTA provided on the ITGV website (http://psd.uohyd.ac.in/itgv/).

## Results

### Whole-genome sequencing of EMS-mutagenized lines

To generate a genome-wide resource for tomato mutants, WGS of 132 independent M_2_M_4_ EMS-mutagenized lines was performed, including their parental cultivar, Arka Vikas (AV) (**Supplementary Figure 1**). In an earlier study (Gupta et al., 2017), the efficacy of the above-mutagenized population was demonstrated, where 55 amplicons belonging to 25 genes derived from a 3-D pooled genomic DNA of 768 M_2_M_2_ EMS-mutagenized plants were sequenced. The high-throughput WGS was carried out on the Illumina HiSeqX platform using 2X 150-bp paired-end sequencing chemistry. The final output generated 4.72 terabytes of raw gene sequence data with 31.5 billion reads. On average, 238 million paired-end reads (35.75 Gb) were obtained for each M_2_M_4_ line with an average sequencing depth of 37.64-fold. The raw reads were filtered (Q30) using fastp software (Chen et al., 2018), and on average, 235 million (range 196–504 million) high-quality paired-end reads were obtained for each line after filtering with an average sequencing depth of 36.5-fold (**Supplementary Table 2A**, range 30.37–78.21-fold). The sequence alignment and variant calling were performed as described in **Supplementary Figure 2**.

### The mutant population exhibited a significant number of A/T>G/C transitions

**Figure 1:**
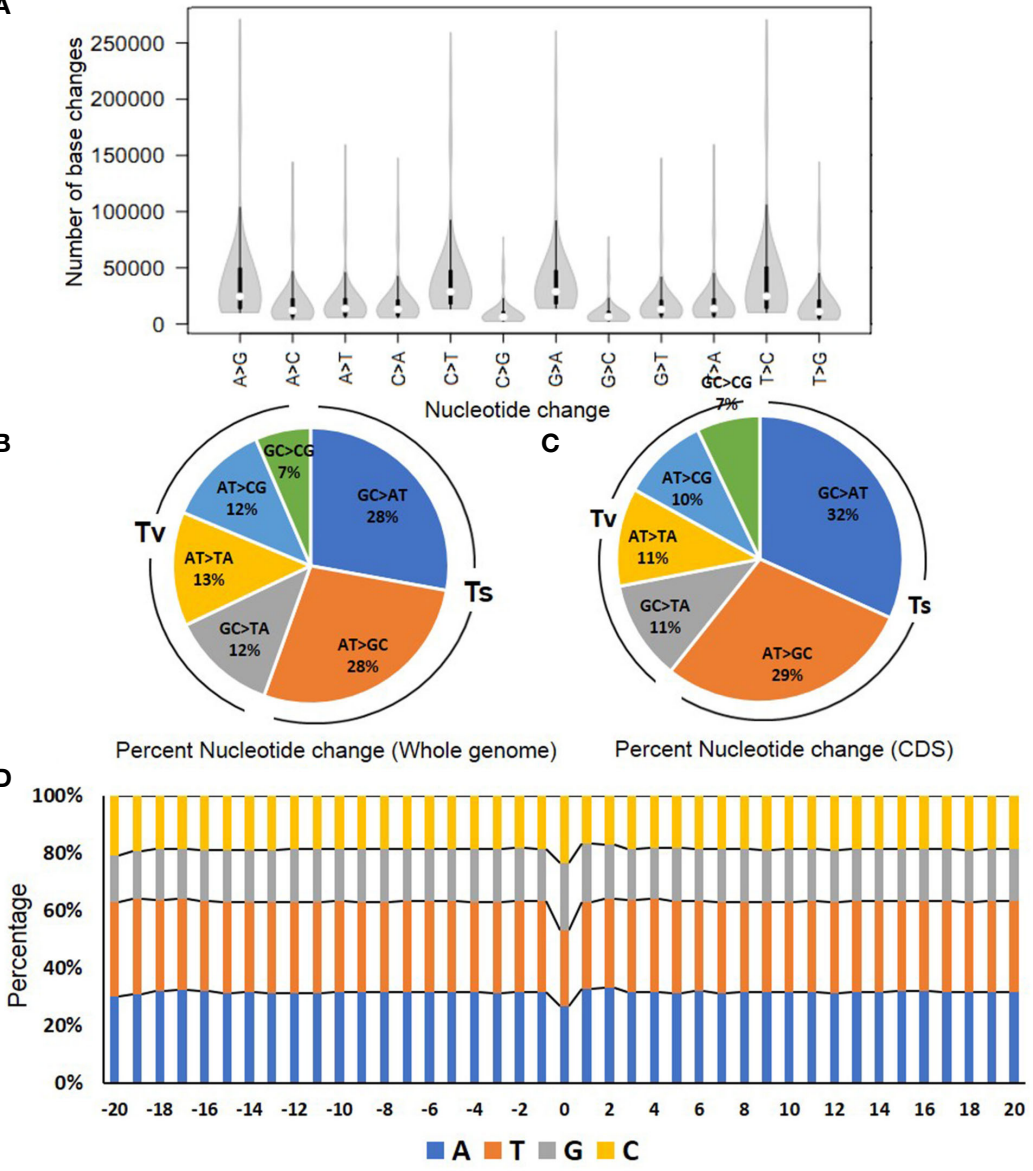
The spectrum of nucleotides changes in the mutagenized population. **(A)** The magnitude of changes in the different nucleotides in the mutagenized population. **(B, C)** The percent nucleotide changes in the whole genome **(B)** and coding sequence (CDS) **(C)**. **(D)** The flanking sequence (20 bp) on either side of the mutations in the genome. The 0 bp represents the site of mutation. The bars depict the percent nucleotides at a given position in the sequence. The colors represent the individual nucleotides with annotations indicated below the graph. For details, see **Supplementary Table 3B**.

After variant filtering to remove artifacts and subtract SNPs present in the reference genome (Arka Vikas), 46.5 million novel variations were detected, comprising approximately 41 million SNPs and 5.5 million short InDels in the analyzed M_2_M_4_ population. Contrary to the expectation, the G/C>A/T transitions were lower, with an average of 27.86%. Surprisingly, the A/T>G/C transitions, which were expected to have a very low frequency, were 27.62%. Nearly the same pattern was reflected for the conversion of individual nucleotides (C➔T, G➔A, A➔G, and T➔C) (**Figure 1A**). The remaining variations comprising transversion (GC>TA, AT>TA, AT>GC, and GC>CG) ranged from 6% to 13%, with C>G and G>C transversions being the lowest (**Figures 1A, B**). This low frequency of C>G and G>C transversions was consistent with other studies, such as EMS-mutagenized rice lines (Yan et al., 2021). To validate the mutations identified, 98 SNPs (71 heterozygous and 27 homozygous) were selected. These SNPs were subjected to Sanger sequencing, and all were confirmed positive (**Supplementary Table 2B**). The above validation confirmed the authenticity of mutations detected by WGS and their transmission to the next generation.

Since we used a doubly mutagenized M_2_M_4_ population, there is a likelihood that the same mutations can exist in multiple individuals. To ascertain how many unique mutations are present and their nucleotide frequency, we calculated unique SNPs in each individual using vcftools (**Supplementary Table 3A**). We identified a total of 48,49,815 unique SNPs in the population (11.80% of the total SNPs). Concurring with earlier analysis, a slightly different pattern of mutation frequency was observed for unique SNPs, with 30.69% being G/C>A/T transitions and 23.30% A/T>G/C transitions.

Purportedly, EMS-induced mutations are strongly biased to G/C>A/T transition (Greene et al., 2003), as EMS preferentially alkylates guanine to *O* ^6^-ethylguanine, which mispairs with T in place of C (Ashburner et al., 2004). To a reduced extent, EMS also mediates AT>GC transition by the alkylation at *O* ^4^ of thymine (Drake and Baltz, 1976) (**Supplementary Figrue 3**). Therefore, we checked whether the digression to high A/T>G/C transition was restricted to a specific region or was omnipresent throughout the genome. The protein coding sequence (CDS) analysis revealed a nearly similar mutation spectrum with 31.74% G/C>A/T and 28.93% A/T>G/C transitions. Correspondingly, the transversion frequency of 7% to 11% in CDS was also closely similar to transversions in the whole genome (**Figure 1C**).

To ascertain if any bias exists in SNPs in the natural population, we chose the Aflitos et al. (2014) study for comparison because it contained SNPs from the present-day cultivars (54) and several wild relatives (30). Interestingly, we found a similar base change bias in the natural population, with 27.31% G/C>A/T and 26.88% A/T>G/C transitions (**Supplementary Figure 4**). The similarity between base changes frequency of EMS-mutant lines and tomato accessions shows that even for EMS mutagenesis, the distribution and transmission of the mutations in the mutagenized progeny are similar to those of natural cultivars.

### Does EMS have any sequence bias?

We next examined whether EMS preferentially induces mutations at genic sites having any particular motif. We analyzed the nucleotide frequencies 20 bp upstream and downstream flanking to the 41 million identified SNPs. Remarkably, we did not find any preferred genic motif or bases upstream or downstream of the mutated site. Nonetheless, our analysis showed that the mutated site had a higher GC percentage (47% of GC-rich region) than the flanking sequences (37% GC-rich region) (**Figure 1D**; **Supplementary Table 3B**).

### Nearly 31% of the population’s induced mutations were unique

The penetrance of mutations showed a wide variation in the population. The mutation frequency in the whole genome ranged from 1/0.45 kb to 1/11.05 kb in mutant lines (**Supplementary Table 4**). Allowing for the tomato genome size of 950 Mb (https://solgenomics.net/about/tomato_project_overview.pl), the average mutation frequency was 1/3.057 kb, considering each line, on average, harbored 311,101 SNPs. The mutation frequency for G/C>A/T transitions in the whole genome was 1/10.958 kb. Considering the mutations localized only in the CDS, the average mutation frequency was 1/6.023 kb, whereas for G/C>A/T transitions, it was 1/16.134 kb (**Supplementary Table 4**). Seemingly, the mutation density of 1/3.057 kb may appear high relative to earlier studies; nonetheless, in Chinese cabbage, even a higher mutation density of 1/0.535 kb was observed (Sun et al., 2022).

As EMS produces random mutations in the genome, we examined the distribution of SNPs in all 12 chromosomes of the population. Line-wise distribution of SNPs revealed that chromosome 11 had the highest SNPs in most lines. Oppositely, chromosomes 4 and 6 had the lowest SNPs (**Supplementary Figure 5**). However, the genome-wide SNP distribution for all lines revealed that chromosomes 0 and 11 were densely populated with SNPs, while chromosomes 3 and 8 were sparsely populated (**Supplementary Table 5**). A similar uneven distribution of SNPs across different chromosomes was observed in *Brassica napus* (Tang et al., 2020).

**Figure 2:**
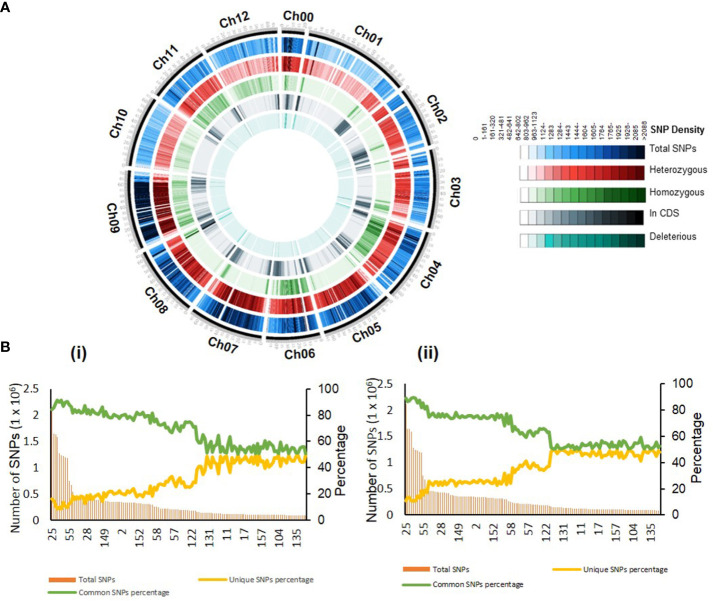
Distribution of the mutations across the genome. **(A)** Circos plot showing the chromosome-wise distribution of different mutations in the mutant population. For details, see **Supplementary Table 5**. **(B)** The unique single-nucleotide polymorphisms (SNPs) present in 132 mutant lines compared to 54 tomato cultivars (i) and 30 wild relatives of tomato (ii). Left axis, total SNPs; right axis, percent unique and common SNPs in mutant lines. Note that percent unique SNPs are lower in the mutant lines with the high density of the mutations. Lines are organized by decreasing the number of total SNPs. For details, see **Supplementary Table 6**.

Both homozygous and heterozygous SNPs showed a random distribution across the genome. Foreseeably, the heterozygous SNPs were numerically higher than the other changes in the nucleotides (**Figure 2A**). We have observed an average of 16.7% (range 0.41%–57.63%) and 19.69% (range 0.85%–54.43%) of homozygous SNPs in the whole genome and gene region, respectively (**Supplementary Table 4**). Seemingly, the occurrence of homozygous mutations is not correlated with the mutation density in mutant lines. For example, line 86 had a mutation density of 1/0.577 kb but had 54.6% homozygous SNPs, whereas line 98 had a mutation density of 1/0.581 kb but had only 0.41% homozygous SNPs (**Supplementary Table 4**).

The exploitation of induced genic polymorphism of the mutant population strongly depends on the uniqueness of the altered SNPs. We ascertained how many induced SNPs were novel than the genic polymorphism present in tomato cultivars. To do this, we compared the 41 million induced SNPs identified in our 132 mutant lines with 539 million naturally existing SNPs reported in 85 tomato lines (Aflitos et al., 2014; Gupta et al., 2020a). Moreover, in the study of Gupta et al. (2020a), we realigned the sequences of 84 tomato lines to SL3.0 assembly, similar to this study. Compared to tomato cultivars and wild relatives, on average, 31.07% (range 8.53%–49.65%) and 35.37% (range 10.66%–49.96%) SNPs in 132 lines were unique in our lines, respectively (**Supplementary Table 6**). Remarkably, the percent of unique SNPs in a mutagenized line is oppositely correlated with the density of mutations. The lines with lower mutation density had the highest percent of unique SNPs and vice versa (**Figure 2B**; **Supplementary Figure 6**).

To ascertain the possibility of cross-pollination or contamination, we compared the unique and common SNPs among nine mutant lines having higher mutation density (>1 million SNPs) to other tomato cultivars and wild type. Our analysis revealed a random distribution of SNPs in the above mutant lines, eliminating the possibility that observed SNP were not due to contamination or cross-pollination events (**Supplementary Table 7**).

### Population is enriched in mutations, impacting protein’s functionality

**Figure 3:**
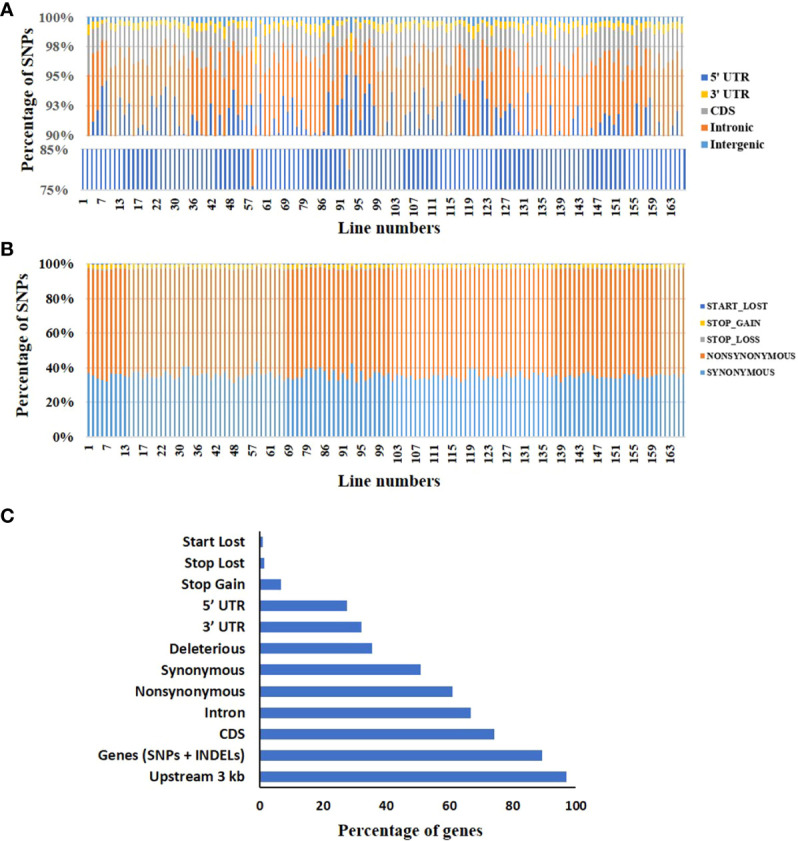
Distribution of mutations in the mutagenized population. **(A)** The relative distribution of mutations in different regions of the tomato genome in the population. For details, see **Supplementary Table 4**. **(B)** The distribution of different functional classes of mutations in the coding sequence (CDS) region of the population. For details, see **Supplementary Table 8A**. **(C)** The relative proportions of different functional categories of mutations, including mutations in promoters and introns.

To assess the impact of the individual mutation on the encoded protein function, the 41 million induced SNPs were annotated using the SIFT4G ITAG3.2 genome reference database (Vaser et al., 2016; Gupta et al., 2020a; https://solgenomics.net). Based on the annotation, 91.5% of SNPs were present in the intergenic region, 5.5% in the intronic region, 2% in the CDS region, and 1% in the UTR region (**Figure 3A**). Out of 2% SNPs in CDS, 36.3% led to synonymous (silent) mutations, 60.8% were non-synonymous (missense) mutations, and 2.9% caused stop-gain/loss and start-loss, leading to truncation of the encoded protein (**Figure 3B**). On average, the CDS in a mutant line contained 2,290 synonymous, 3,834 non-synonymous, 32 stop-loss, 136 stop-gain, and 16.5 start-loss mutations (**Supplementary Table 8A**). Additionally, the mutant lines also had several deletions comprising both frameshift and non-frameshift InDels. On average, a mutant line harbored 694 frameshift insertions and 601 frameshift deletions (**Supplementary Table 8B**). We also found 454 unique mutations in 156 miRNAs, with heterozygous mutations (79%) constituting the majority (**Supplementary Table 9**).

In the 132 mutant lines, 89.4% of genes harbored at least one SNP/InDel, with 74.2% having SNP/s in the CDS region. Among the SNPs in the CDS, 60.9% of genes harbored non-synonymous mutations (**Figure 3B**). Our analysis revealed that almost 97% of genes had at least one SNP/s in the 3 kb upstream region of the gene (**Figure 3C**). Among these mutations, 35.6% of genes had mutations with SIFT score <0.05 and therefore were predicted to be deleterious (Ng and Henikoff, 2003; Kumar et al., 2009) (**Supplementary Table 10**). Consistent with the random nature, these deleterious mutations were distributed across the genome. A limitation of the SIFT is that it is geared to predict the effect of a single amino acid change on the protein function. The SIFT does not predict the influence of the start-loss, stop-gain, and stop-loss variants on the protein function. Indubitably, these variants can also be deleterious or affect the protein’s function. These variants were present in the range of 1% to 7% of the genes in the mutant population. The effect of the mutations in the intergenic region, particularly in the promoter, is not directly quantifiable like the ones affecting the amino acids (**Figure 3C**). Nonetheless, these mutations are also important, as the *cis*-regulatory region/promoter region variation can modulate gene expression and create trait diversity.

### Effect of mutations on individual codons and amino acids

**Figure 4:**
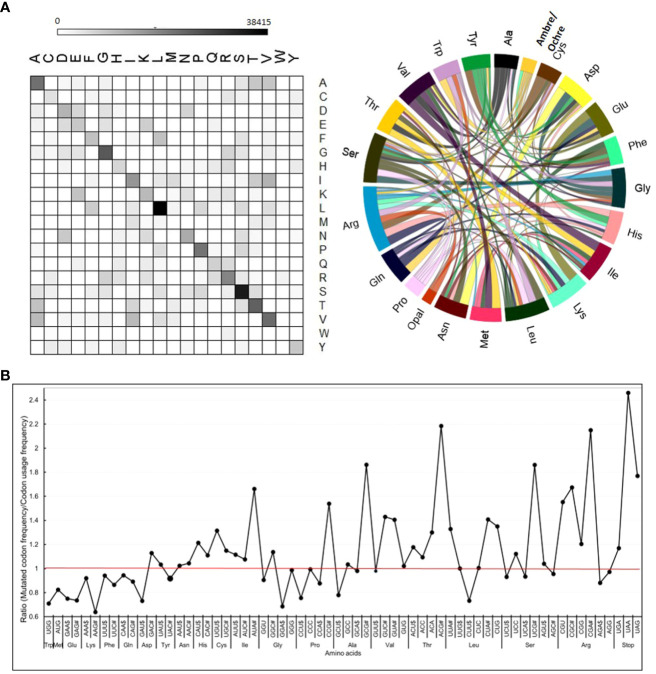
Frequency of mutations in different codons and amino acids. **(A)** Relative frequency of changes in individual amino acids in mutagenized population. All 168 possible amino acid changes were observed with varying frequencies. For details, see **Supplementary Tables 12**, **15**. **(B)** The ratio between the frequency of a mutated codon in the mutagenized population and its normal frequency in tomato. The most preferred codon, as per the tomato codon usage table (https://solgenomics.net/documents/misc/codon_usage/codon_usage_data/l_esculentum_codon_usage_table.txt), is marked with the $ dollar sign, and the least preferred codon is marked as # hashtag after the codon letter in the graph. The individual ratios are given in **Supplementary Table 12**.

The degeneracy of the genetic code protects the genome against the mutation loads caused by spontaneous mutations. Notwithstanding the genetic code degeneracy, nearly 62.8% of CDS mutations were non-synonymous. Among the non-synonymous changes, 4% belonged to the valine to isoleucine and vice versa (V/I and I/V) and alanine to valine (A/V). Among the 37.2% synonymous mutations, the major changes were leucine to leucine (L/L) and serine to serine (S/S) (**Figure 4A**; **Supplementary Table 11**). These two changes comprised approximately 9% of total amino acid substitutions. A high degree of synonymous changes in leucine and serine was expected, as these two amino acids were encoded by six codons.

We compared whether the mutagenicity of codons had any relationship to their usage in a protein by comparing it with codon usage frequency in tomatoes. We checked it by calculating the ratio between the frequency of a mutated codon and its usage in wild-type tomatoes. We considered the most used codon for an amino acid as the preferred codon. Interestingly, for amino acids having a higher degeneracy of three to four codons, the preferred codons were the least prone to mutagenesis (ratio ≤ 1). Conversely, the least preferred codons were the most prone to mutagenesis (ratio ≥ 1). Interestingly, even for the stop codons, the ratio for the most preferred codon, UGG, was lower (1.04), while less preferred codons (UAA- 1.86 and UAG- 1.82) had higher ratios (**Figure 4B**; **Supplementary Table 12**). Remarkably, the analysis of tomato cultivars also showed that the least preferred codon was the most prone to mutation, with a near overlap in the pattern of mutagenized lines and cultivars (**Supplementary Figure 7**, **Supplementary Table 13**).

Markedly, the amino acids encoded by two codons did not show the above mutagenicity bias, as the ratios were nearly similar and largely remained ≤1. Comparing the ratio of the frequency of mutated codons with that of codon usage revealed that methionine (0.73) and tryptophan (0.77) were the least prone to mutagenesis. Since methionine and tryptophan lack codon degeneracy, any mutation leads to a non-synonymous change.

The dominance of transitions over transversions in the overall mutational spectrum was strongly seen in the CDS mutations. The theoretically predicted ratio of mutations for an amino acid was calculated with the assumption that mutations are random and strongly deviate from the observed mutations (**Supplementary Table 14**). For illustration, the mutation from valine to alanine, where the middle codon changes from T to C, signifying transition, has a ratio > 1 (24.49/16.66 = 1.46). In contrast, the mutation from valine to glycine, where the middle codon changes from T to G, signifying transversion, has a ratio < 1 (8.39/16.66 = 0.50). The above difference in pattern between theoretical and actual distributions of transitions or transversions was consistently observed for most amino acids (**Supplementary Table 14**). The average ratio of transitions (1.80) was nearly 2.38-fold higher than that of transversions (0.756).

### Housekeeping genes are recalcitrant to EMS mutagenesis

**Figure 5:**
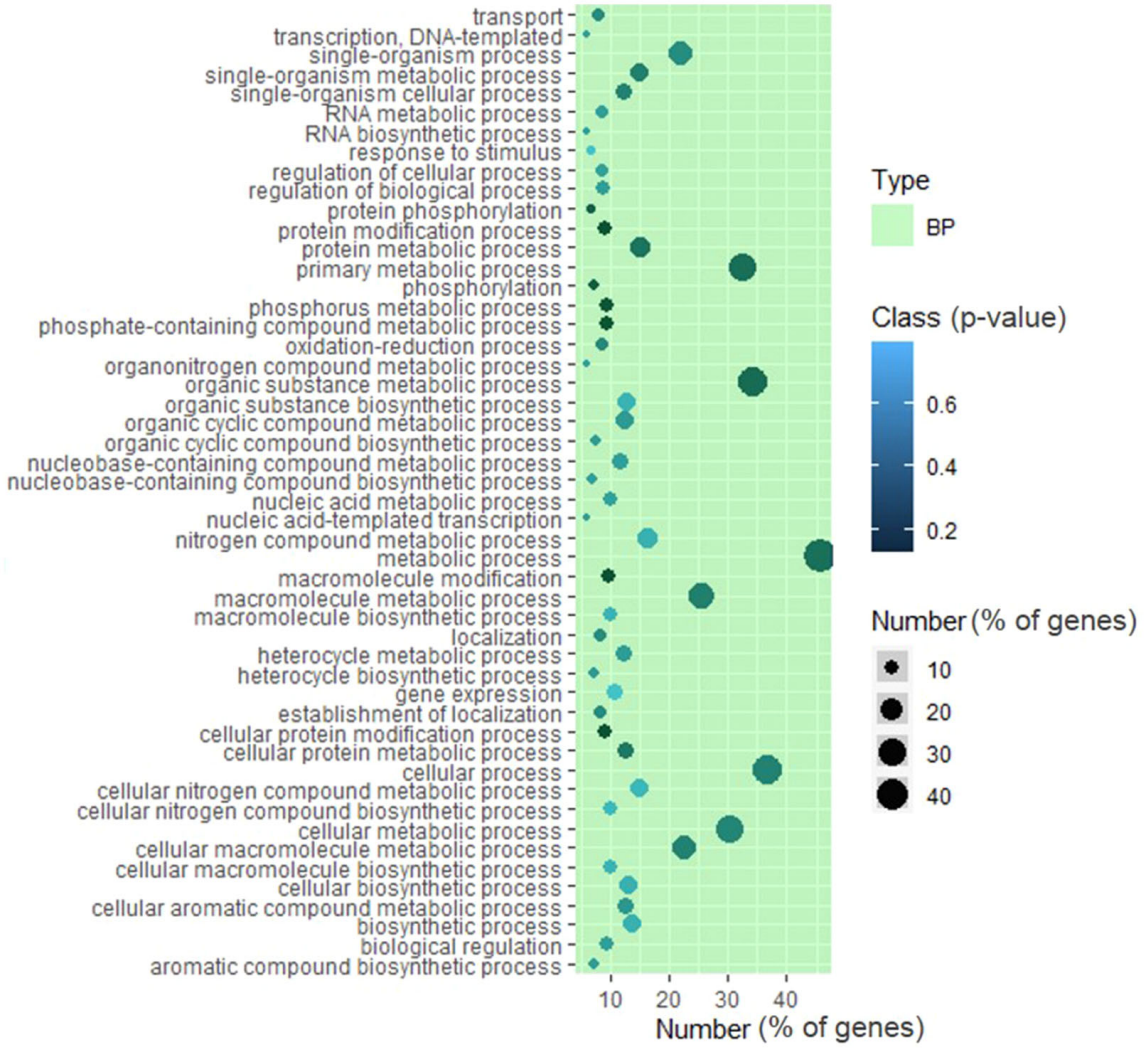
Distribution of mutations in different Gene Ontology (GO) categories and on the metabolic pathway. Top 50 GO categories [biological process (BP)] with the high frequency of mutations. Note that in none of the GO categories, the percent mutations exceed 50%. For details, see **Supplementary Table 15**.

Gene Ontology (GO) analysis revealed that a broad range of the categories bore non-synonymous and synonymous mutations, including mutations in the UTR (**Figure 5**; **Supplementary Figure 8**; **Supplementary Table 15**). In the mutant population, nearly 40% of genes bore no non-synonymous mutations (**Figure 3C**). Across the different GO categories, the absence of mutations was mainly in housekeeping genes related to the gamut of the essential cellular processes. For instance, no mutations were detected in GO categories such as tetrahydrobiopterin synthesis, t-RNA 3′ end processing, mitochondrial respiratory chain complex, and ATPase activity regulation.

In mammals, approximately 35% of genes are essential for survival and housekeeping (Dickinson et al., 2016), and the same is likely for plants. In *Plasmodium falciparum*, large-scale insertional mutagenesis revealed that nearly 50% of genes are essential for optimal growth (Zhang et al., 2018). Since many GO categories showed no non-synonymous mutations, these genes are presumably essential for tomatoes. Our results are in conformity with large-scale mutagenesis in *Caenorhabditis elegans*, where too the essential genes lacked mutated alleles (Thompson et al., 2013). The lack of mutations in housekeeping or essential genes in tomatoes indicated that these genes are largely recalcitrant to mutagenesis. It can be construed that, similar to spontaneous mutations in nature (Monroe et al., 2022), the EMS-induced mutations in essential genes are subjected to strong purifying selection and elimination. It remains to be determined whether the epigenomic state of essential genes, as observed in *Arabidopsis*, reduces the rate of mutations (Monroe et al., 2022). It may be noted that our GO analysis represents only the coding mutations. Nonetheless, the mutations in the non-coding region may also influence the function and/or expression of essential housekeeping genes.

### Metabolic pathways affected by mutations

To specifically evaluate the influence of mutations on a broader scale, we mapped the genes predicted by SIFT to have a loss of function (LOF) on the tomato metabolic pathway. Out of the 7,991 genes currently assigned in LycoCyc to the tomato metabolic pathway (https://solcyc.solgenomics.net/organism-summary?object=LYCO, ver. 3.8. ITAG3.2), 2,861 genes were mapped with LOF (**Supplementary Figure 9A**). The mapping of all non-synonymous mutations on tomato metabolic pathways highlighted that most pathways had one or more mutant genes that may affect their operation (**Supplementary Figure 9B**). Thus, our mutant resource contained novel mutations affecting a range of metabolic pathways and has the potential for breeding and functional genomics.

### Phenotyping and metabolic mapping of the population

The immediate progeny of 132 EMS-mutagenized lines was visually phenotyped in three different growing seasons for morphological changes. Extensive variations were found in plant height, branching, leaf shapes, fruit size, and sets. The variations were digitally cataloged (**Supplementary Figure 10**), and phenotypes of mutant lines can be assessed online as a link in the database (http://psd.uohyd.ac.in/itgv/). Among these lines, a mutant line RM277A (line 047) with potato leaf phenotype was analyzed and revealed a mutation in a MYB transcription factor (**Supplementary Figure 11**). The above mutation resides in potato leaf locus (locus *c*) (Solyc06g074910) (predicted deleterious by SIFT). The above mutation is a novel locus *c* allele. It adds to other reported potato leaf-type mutants caused by transposon insertion in the *c* locus (Busch et al., 2011) and P42R change in an heirloom tomato (Rowland et al., 2020). Crosschecking our database revealed a non-synonymous mutation Y164H in the *c* locus of line 79 and line 81 (**Supplementary Figure 11**). However, Y164H mutation was not predicted deleterious by SIFT, and consistent with SIFT prediction, the mutant lines did not display potato leaf phenotype.

**Figure 6:**
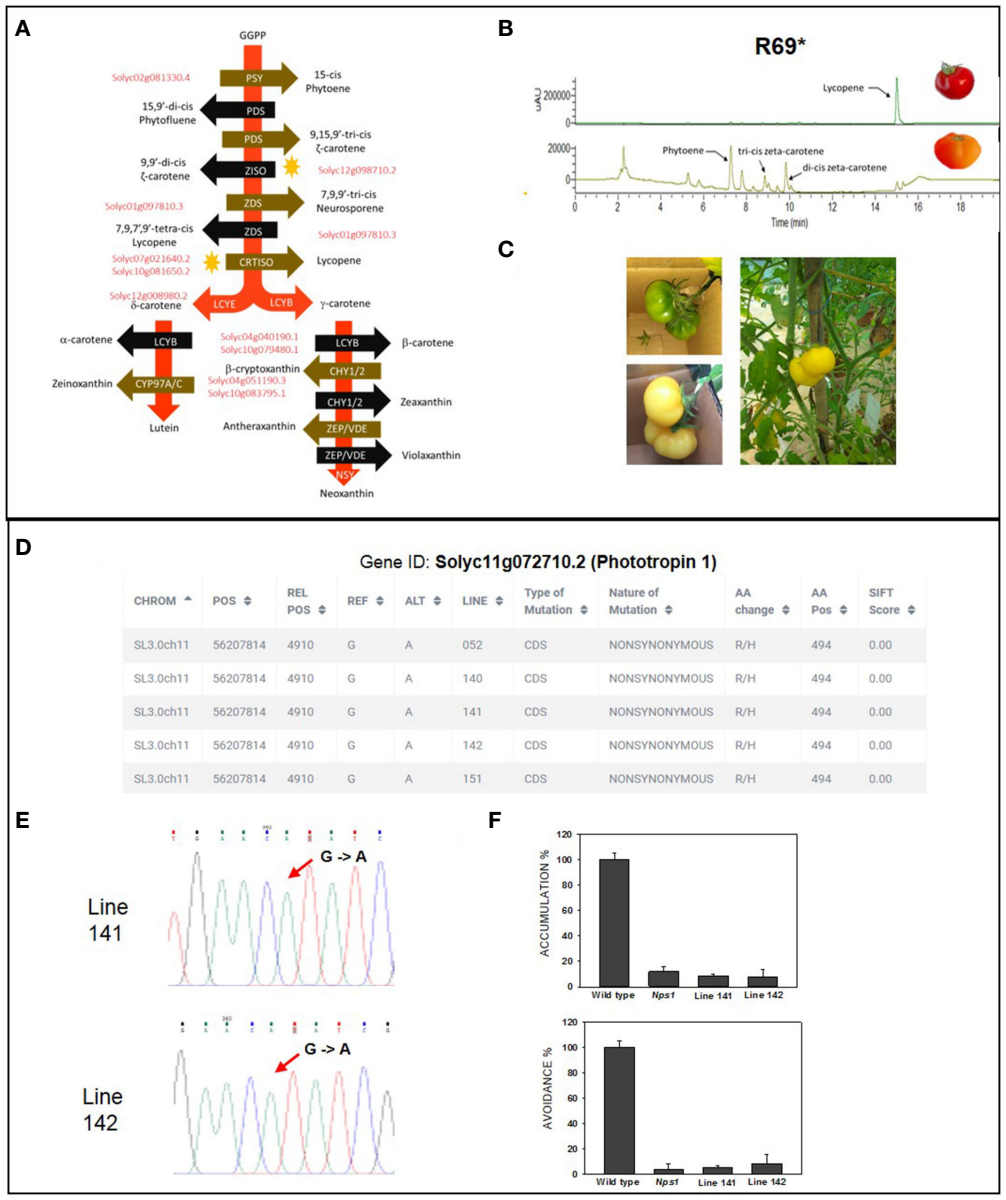
Validation of light signaling and carotenoid biosynthesis mutants. **(A)** Carotenoid biosynthesis pathway in tomato showing the 11 deleterious mutations present in the population (gene IDs are in red, and *ziso* mutation is marked with ↑). **(B)** The chromatograms show the loss of lycopene formation in the *ziso* mutant (R69*). Note that, unlike the wild type, the mutant forms little lycopene but shows tri-*cis*-ζ-carotene and di-*cis*-ζ-carotene (bottom panel), which are present in traces in the wild type (top panel). **(C)** The loss of ζ-carotene accumulation in *ziso* fruit shielded from light. The fruit was shielded from light at the mature green stage, and photographs were taken after 12 days. The absence of light massively reduced the formation of tri-*cis*-ζ-carotene and di-*cis*-ζ-carotene in fruits. **(D)** Screenshot from Induced Tomato Genomic Variations (ITGV) database showing the lines containing R494H substitution in *phototropin1* gene. Note: In recent ITAG3.2 annotation, the *Nps1* mutation is located at R494H. **(E)** The homozygosity of mutations in lines 141 and 142 were validated by Sanger sequencing. **(F)** The chloroplast relocation response in leaves of mutant lines 141 and 142. Both lines show near-total loss of chloroplast accumulation and avoidance response, similar to the *Nps1* mutant. Abbreviations: PSY, phytoene synthase; PDS, phytoene desaturase; ZISO, ζ-carotene isomerase; ZDS, ζ-carotene desaturase; CRTISO, carotenoid isomerase; LCYE, lycopene ϵ-cyclase; LCYB, lycopene β-cyclase; CYP97A/C, cytochrome P450 monooxygenase; CHY1/2, β-carotene hydroxylase; ZEP, zeaxanthin epoxidase, VDE, violaxanthin epoxidase; NSY, neoxanthin synthase.

We also specifically examined the distribution of mutated genes on three important metabolic pathways: tetrahydrofolate biosynthesis, carotenoid biosynthesis, and plant photoreceptors/circadian regulation. We found three deleterious mutations affecting the tetrahydrofolate pathway (**Supplementary Figure 12**), 15 deleterious mutations in the light-signaling pathway (**Supplementary Figure 13**), and 11 deleterious mutations in the carotenoid biosynthetic pathway (**Figure 6A**). To validate whether a SIFT-predicted deleterious mutation alters the phenotype from the above mutations, we selected two mutants: one from the carotenoid pathway and one from the photoreceptors.

### Characterization of a ζ-carotene isomerase mutant

We validated the presence of a deleterious homozygous mutation in ζ-carotene isomerase (*ZISO*) gene in the B1 line, harboring a stop codon at the 69th amino acid position. All progeny plants from the B1 line bore orangish-red fruits, a characteristic shared with other reported *ziso* mutants of tomato (Fantini et al., 2013). Usually, the mutants compromised in the early steps of carotenoid biosynthesis are lethal, except ZISO and carotene isomerase (*CRTISO*), which execute carotenoid isomerization. Since the carotenoid isomerization catalyzed by ZISO and CRTISO can also be catalyzed by light, the photosynthetic activity in green leaves is not comprised in these mutants. However, the light fails to penetrate the deeper tissue layers of tomato fruits. Therefore, carotenoid biosynthesis becomes stalled at the conversion of tri-*cis*-ζ-carotene to di-*cis*-ζ-carotene in *ZISO* mutant, resulting in orangish-red fruits.

Consistent with the *ziso* mutant being compromised in carotenoid isomerization, the mutant fruits had higher amounts of tri-*cis*-ζ-carotene and di-*cis*-ζ-carotene, confirming that the mutant was indeed comprised in ZISO activity. The tri-*cis*-ζ-carotene and di-*cis*-ζ-carotene do not accumulate in wild-type fruits, as light along with ZISO and CRTISO enzymes convert them to downstream carotenoids, mainly lycopene (**Figure 6B**). To reduce light-mediated isomerization of carotenoids, we covered on-vine the mature-green *ziso* mutant fruits with black sheets. The enclosed fruits were yellow-colored and had reduced levels of tri-*cis*-ζ-carotene, di-*cis*-ζ-carotene, and low lycopene, confirming that the covering of fruits substantially blocked light-mediated carotenoid isomerization and validated the functional loss of ZISO activity (**Figure 6C**).

### Characterization of a phototropin1 mutant

In tomatoes, the mutation in the phototropin1 gene changing arginine to histidine at 495th amino acid (Arg495His, now revised to R494H as per ITAG 3.2) dominantly blocks the light-induced chloroplast accumulation and avoidance response in leaves (Sharma et al., 2014). We found the same genic variant in five mutant lines, viz., 052, 140, 141, 142, and 151. Two mutant lines, 141 and 142, had the mutation in the homozygous state. We examined the chloroplast relocation responses in homozygous 141 and 142 lines, wild type (Arka Vikas), and *Nps1* mutant. Consistent with the dominant-negative effect of Arg494His mutation, the chloroplast relocation response in 141 and 142 lines was blocked similarly to the *Nps1* mutant and its backcrossed progeny (**Figures 6D–F**; **Supplementary Figure 14**).

### Web-searchable access to mutations

**Figure 7:**
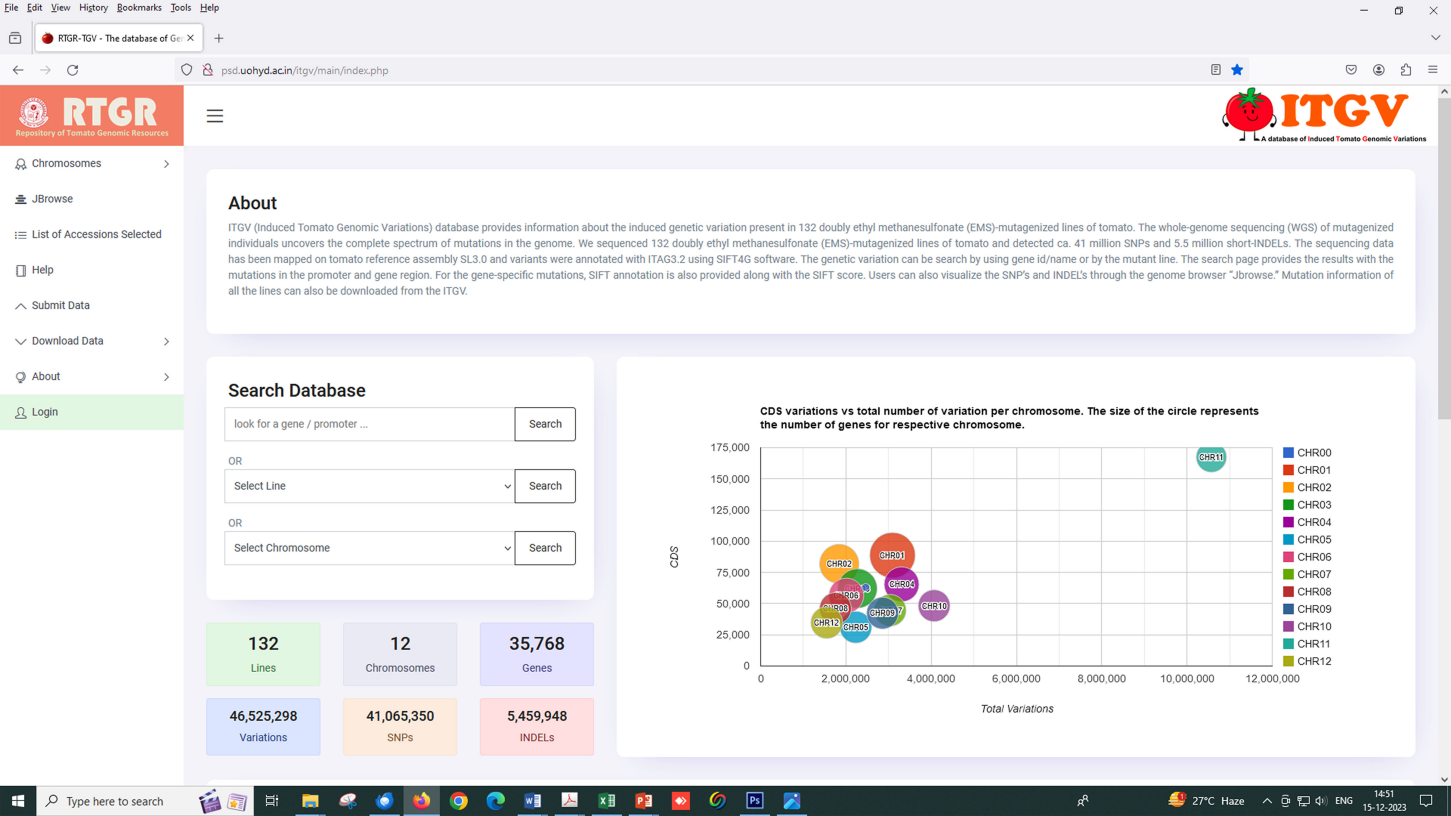
Screenshot of the tomato mutant database for variant visualization, variant information, and annotation (http://psd.uohyd.ac.in/itgv/).

To make this comprehensive mutant resource and its corresponding data available to the public, we made an open-access database called the ITGV database (http://psd.uohyd.ac.in/itgv/). The users can search the ITGV database by gene ID/name or mutant line. The search page provides the results with the promoter and gene region mutations. For the gene-specific mutations, SIFT annotation is also provided along with the SIFT score (**Figure 7**). Users can also visualize the SNPs and InDels through the genome browser “Jbrowse”. Mutation information of all the lines can also be downloaded from the ITGV. The users can request mutant seeds, the details of which are provided on the ITGV website.

## Discussion

### WGS uncovered a high mutation density in tomato

In this study, we sequenced and cataloged a doubly EMS-mutagenized tomato mutant population. The genome-wide analysis of mutations brought forth an unexpected aspect discordant with earlier reports. The observed average mutation density of 1/3.057 kb is many-fold higher than earlier reports. Overall, the magnitude of mutations was >100,000 per mutant line. The higher mutation density may have resulted from the usage of the population subjected to two independent rounds of mutagenesis (120 mM EMS). It is reported in barley (Jiang et al., 2022) and tomato (Minoia et al., 2010) that the mutation frequency increases with a higher dosage of EMS. The high mutation density also entails that a relatively small population is needed to mutate all the genes in tomatoes. The analysis of tomato EMS-mutagenized TILLING mutants revealed a mutation density range from 1/322 kb to 1/1,710 kb (Gady et al., 2009; Minoia et al., 2010; Piron et al., 2010; Okabe et al., 2011; Okabe et al., 2013; Okabe and Ariizumi, 2016). In an earlier TILLING study, we found a mutation density of 1/367 kb in the Arka Vikas cultivar (Gupta et al., 2017). Seemingly, the WGS, being more robust, uncovered the real quantum of mutations in our population. The higher mutation density in tomatoes is also contrary to the notion that only polyploid species such as wheat (Uauy et al., 2009) or polyploid *Arabidopsis* (Tsai et al., 2013) can tolerate the mutation load owing to extra copies of the genome.

It remains possible that in conventional TILLING, due to the widely varying efficiency of CEL-I to cleave different mismatches (C/C ≥C/A ∼ C/T, G/G >A/C ∼ A/A ∼ T/C > T/G ∼ G/T ∼ G/A ∼ A/G >T/T; Oleykowski et al., 1998), the bulk of mutations escape detection. Similarly, the mutations can escape detection in NGS-based TILLING due to the inadequacy of available software to precisely identify mutations in 64X DNA pooling (Gupta et al., 2017). In WGS using individual mutant lines, the variant detection in a mutagenized population is more robust, particularly with sequencing coverage higher than 15X (Thompson et al., 2013; Krasileva et al., 2017). The higher sequencing depth of 30X allowed us to detect a larger number of variants ranging from 1/0.45 kb to 1/11.05 kb in mutant lines.

Unlike other studies, our study’s mutation frequency was higher, as our analysis included all SNPs obtained at a higher sequencing depth of 30X and used a doubly EMS-mutagenized population. In rapeseed, the frequency of G/C>A/T mutations was 1/23.6 kb, whereas when the complete mutation spectrum was included, the frequency was much higher at 1/13.3 kb (Jhingan et al., 2023). Comparing the mutation frequency of 1/16.134 kb in this study to that of 1/23.6 kb reported in rapeseed (Jhingan et al., 2023) for G/C>A/T mutations, our mutation frequency was close to that of other reports. Moreover, the analysis such as in maize, rapeseed (four lines, 20X, Jhingan et al., 2023), and wheat (8–10 lines, 11.63X, Wang et al., 2023) was carried out using the DNA pooled from different M_2_/M_3_ plants at lower sequencing depths, which may have hindered the robust mutation detection.

Using WES at 14.82X coverage for exons (intron 9.48X, promoter 7.35X coverage) in maize, mutation density was 1/48 kb (Lu et al., 2018). Since WES was carried out in M_1_ maize plants obtained from mutagenized pollens, the mutation frequency (1/48 kb) was practically half, as only a single haploid genome was mutagenized. In the Cadenza wheat M_2_ population, WES at 29.01X coverage revealed an average mutation frequency of 1/30.3 kb (Krasileva et al., 2017). In KN9204 wheat, WES at 11.63X coverage had an average mutation frequency of 1/50.2 kb (Wang et al., 2023). In maize, like in humans (Belkadi et al., 2015), a comparison between WES and WGS revealed that WGS is more robust in mutation detection than WES, even in the exome. Compared to the WES of the Bp001 mutant maize line, the WGS detected nearly twofold higher mutations in the same exonic region (Lu et al., 2018). Recently, a very high mutation density of 1/0.535 kb was reported in Chinese cabbage (Sun et al., 2022), much higher than the mutation density (1/3.057 kb) observed in our population. Thus, it can be inferred that using WGS with ≥30X coverage allowed the detection of a far higher mutation density than other studies.

Interestingly, the lines bearing high mutation density also share a high percentage of SNPs present in 53 tomato cultivars (Aflitos et al., 2014; Gupta et al., 2020a). It can be construed that the SNPs present in tomato cultivars arose by spontaneous mutations and were not subjected to purifying selection (Charlesworth et al., 1993). In all likelihood, the EMS mutations in these SNPs shared with tomato cultivars had a higher probability of being retained and passed to the next generation. Considering that unique SNPs at maximum were <50% underscores the importance of purifying selection.

The high proportion of heterozygous mutations observed in our population is likely a result of double mutagenesis and a higher dose of EMS. Several studies (Greene et al., 2003; Martín et al., 2009; Li et al., 2017; Hussain et al., 2018; Schreiber et al., 2019) have reported that mutant populations generally exhibit more than twofold higher heterozygous mutations compared to homozygous mutations. In Chinese cabbage, which, like tomato, has a high mutation density, the homozygous mutations were 21.29%, 24.02%, 28.91%, and 29.35% in the M_2_, M_3_, M_4_, and M_5_ generations, respectively (Sun et al., 2022). Apparently, the advancement of mutant progeny only resulted in a minor enrichment of homozygous mutations.

### EMS also caused AT>GC transitions

Several studies using WES or WGS restricted mutation analysis to GC>AT transitions (Addo-Quaye et al., 2017; Lu et al., 2018), though non-canonical mutations other than GC>AT contributed to 45%, 51%, 54.9%, and 73.02% of total SNPs in rapeseed (Jhingan et al., 2023), sorghum (Addo-Quaye et al., 2017), maize (Lu et al., 2018), and wheat (Hussain et al., 2018), respectively. In sorghum, 51% and 62.8% of non-GC>AT SNPs were excluded from the final analysis, assuming that these were artifacts (Addo-Quaye et al., 2017; Addo-Quaye et al., 2018). Likewise, Jiang et al. (2022) considered only GC>AT transitions in barley. In sunflowers, only 26% of mutations aligned with the canonical GC>AT transitions, and the remaining were non-canonical mutations (Fanelli et al., 2021). Considering that EMS induces a substantial percentage of non-canonical mutations, it is incorrect to exclude them from the analysis. The detection of non-canonical EMS mutations may be peevish, but these mutations likely arise due to the secondary effect of EMS mutagenesis and are heritable (Henry et al., 2014).

In this study, WGS revealed that GC>AT transitions comprised only 30% of overall unique SNPs in the mutant population. In an earlier study, the amplicon sequencing of the progenitor of our mutant population revealed 65% GC>AT transitions (Gupta et al., 2017). Considering that we used a doubly mutagenized EMS population [120 mM (1.5%)], the remutagenesis likely reduced the frequency of GC>AT transitions. A seemingly higher dosage of EMS reduces GC>AT transition in tomatoes, as reported by Minoia et al. (2010), whereupon increase from 0.7% to 1% EMS reduced GC>AT transition from 60% to 28%. In plants and even in EMS-mutagenized *C. elegans* strains, the GC>AT transition frequency was 66% (Sarin et al., 2010). The induced non-canonical mutations detected in our population substantially overlap with SNPs present in 53 tomato cultivars (Aflitos et al., 2014; Gupta et al., 2020a). Since the SNPs have no nucleotide bias, the above overlap emphasizes that non-canonical mutations, other than GC>AT transitions, resulted from EMS treatment.

The selection of GC>AT transitions as the genuine mutations is based on the premise that EMS introduces an alkyl group at *O* ^6^-guanine, leading to G mispairing to T during DNA replication. It is believed that, unlike in humans, *O* ^6^-alkylguanine is not repaired in plants, as the plants reportedly lack *O* ^6^-alkylguanine-DNA alkyltransferase activity (Leitao, 2011; Pegg, 2011). It remains to be determined whether the reported removal of *O* ^6^-alkylguanine in *Vicia faba* root tips (Baranczewski et al., 1997a; Baranczewski et al., 1997b) uses an alternate mechanism such as base excision repair (Manova and Gruszka, 2015).

The EMS-driven non-GC>AT transitions are considered the side effects of mutagenesis and often are not included as potential mutations (Addo-Quaye et al., 2017; Addo-Quaye et al., 2018; Lu et al., 2018). It may be imprudent to ignore these mutations, as WGS does not have a nucleotide bias, and at high coverage such as 30X used in our study, the error rate is extremely low. Consistent with being genuine mutations, the Sanger sequencing validated both canonical and non-canonical mutations. EMS treatment can induce secondary mutations due to errors in DNA repair, including DNA breakage, as evident by the presence of InDels in the mutant population. In crop plants, the frequency of mutations other than GC>AT widely varies with 10% in *Arabidopsis* (Greene et al., 2003), 30% in rice (Till et al., 2007; Henry et al., 2014), 51% in sorghum (Addo-Quaye et al., 2017), and 54.9% in maize (Lu et al., 2018). The WGS of EMS-mutagenized *Toxoplasma* revealed that ~74% of mutations were in the A/T base pair (Farrell et al., 2014). The WGS of EMS-treated MicroTom lines revealed 39%–76% GC>AT transition (Shirasawa et al., 2016), while the WES of 95 tomato mutants displayed only 20.7% GC>AT transitions (Yano et al., 2019). We believe that the wide difference in GC>AT transition is a species-specific phenomenon perhaps related to the difference in the repair efficiency.

### *O* ^4^-Alkyl-thymine may be the causative agent for AT>GC transitions

The AT>GC transition arises from EMS-mediated alkylation at *O* ^4^ of thymine (Drake and Baltz, 1976), which can mispair during DNA replication leading to mutagenicity. In conformity with the above, in *Escherichia coli* and human cell lines, the incorporation of *O* ^4^-alkyl-thymine in DNA results in a large number of T➔C mutations (Wang et al., 2015; Wu et al., 2016). It is plausible that in plants, the formation of *O* ^4^-alkyl-thymine may lead to T➔C mutations during DNA replication.

Contextually, it is likely that the higher degree of A/T>G/C transitions observed in this study reflects the mutagenicity of *O* ^4^-alkyl-thymine residues. In this and an earlier study (Gupta et al., 2017), the Sanger sequencing validated both GC>AT and AT>GC transitions with nearly the same frequency. Thus, it can be surmised that parallel to GC>AT transitions, EMS also induces the AT>GC transition, albeit at a lower frequency. Compared to EMS-mediated *O* ^6^-alkyl-guanine formation, the formation of *O* ^4^-alkyl-thymine is much less (Leitao, 2011). The lower frequency of AT>GC transition is consistent with the said EMS efficiency and consistent with the increase in AT>GC transitions with higher EMS dosage to tomato (Minoia et al., 2010).

### The least preferred synonymous codons are most prone to mutagenesis

The degeneracy of genetic code implies that the synonymous codons for an amino acid vary widely in frequency (Ikemura, 1985). It is believed that among the highly degenerate codons, some codons are preferred over others because they are translated more efficiently and accurately (Hershberg and Petrov, 2008; Hershberg and Petrov, 2009). Another view is that evolutionary selection favors preferred codons over other minor codons, while mutational pressure and genetic drift allow the minor codons to persist (Bulmer, 1991). Contrarily, in moss, it was suggested that weak natural selection for translational efficiency shapes the codon bias rather than the mutational bias (Stenøien, 2005). The codon usage is not limited to genes. A recent report using the Ramachandran plot demonstrated an association between synonymous codon usage and the structure of the translated amino acids (Rosenberg et al., 2022). Our results bring a different paradigm to the codon usage bias. It can be construed that the preferred codons are least mutagenic to EMS, as these are the main codons for translating critical proteins such as ribosomal proteins, elongation factors, and t-RNA. Conversely, the least preferred codon has the highest propensity to mutagenize. Seemingly, mutation bias plays a role in selecting the preferred codon in tomato. The codon most preferred in natural selection is strongly disfavored for mutations.

The classification of mutations across the genetic code table reveals that the transversions generate more non-synonymous mutations than the transitions. Nevertheless, transitions have a ratio higher than the theoretically possible ratio at mutated amino acid levels, while transversions show a converse pattern. Considering that the non-synonymous transitions are purported to be less deleterious (Zhang, 2000; Lyons and Lauring, 2017), the transitions have more likelihood of being retained in progeny. The less deleterious effect of transitions could be related to their influence on protein function, as transitions do not cause drastic changes in amino acid physicochemical properties such as polarity, charge, and size (Zhang, 2000).

### Nearly 3,000 deleterious mutants were mapped on different metabolic pathways

The repertoire of mutations identified in our study is valuable for identifying the function of unassigned as well as known genes. In particular, the mutations in genes modulating metabolic pathways can be used singly or in combination to examine the influence on metabolome/proteome and plant phenotype. Based on the potato leaf phenotype, we validated the above mutation to be residing in locus *c* bearing C50R change. Likewise, in maize (Lu et al., 2018) and sorghum (Addo-Quaye et al., 2017), mutations in gibberellin (GA) biosynthesis were preferentially selected to validate the mutations identified by WES and WGS. The above mutants compromised in the GA biosynthesis pathway were dwarf due to a reduction in GA levels; thereby, the phenotype was rescued by GA. We also selected two highly penetrant mutants by metabolic pathway mapping, viz., the ZISO enzyme that executes carotenoid isomerization, a key step in carotenoid biosynthesis, and phototropin1, which mediates chloroplast accumulation to optimize photosynthesis under weak light.

Though a single-copy gene encodes ZISO, the knockout mutation is not lethal, as carotenoid isomerization can also be photochemically carried out by light. Since insufficient light penetrates the deeper layers of fruits, *ziso* mutant accumulates di- and tri-*cis*-ζ-carotene, while in the wild type, it is below the detection limits. Consequently, the lycopene level in the *ziso* fruits is considerably reduced than wild type. Further, in covered *ziso* fruits, lycopene accumulation is massively reduced due to blockage in light, which supports the role of ZISO in carotenoid isomerization.

The dominant-negative *Nps1* mutation (Arg494His) in phototropin1 blocks chloroplasts’ relocation responses in the mutant leaves. The chloroplasts stay at the bottom of mesophyll cells and do not move toward weak light or move away from strong light (Sharma et al., 2014). Like the *Nps1* mutant, two independent homozygous phototropin1 mutant (Arg494His) lines lacked the chloroplast relocation response. The loss of chloroplast accumulation in BC_1_F_2_ lines further supports that Arg494His mutation has similar dominant-negative action in Arka Vikas as reported for *Nps1* in Ailsa Craig’s background.

Screening our mutant resource using new and uncharacterized genes may uncover the phenotypes not covered by the forward genetics, particularly those leading to metabolic changes that may not have a phenotype. To that effect, we provide a cellular overview of the metabolic pathway where mutations may be affecting steps of a given pathway. In addition, the mutant resource provides scope for silencing a particular pathway by combining the mutations from different lines into the wild-type background.

### WGS provides a broader repertoire of mutants than WES

Unlike *Arabidopsis*, the function of the majority of genes in tomatoes remains unexplored due to the lack of mutants and t-DNA/transposon-tagged lines. The availability of a gene-indexed mutation database bridges this gap for tomato functional genomic analysis (http://psd.uohyd.ac.in/tgv/). The high density of mutations in our population, in essence, provides multiple alleles for several genes. These allelic variants can be used to analyze a selected gene’s function or a group of genes. The potential effect of mutations on the protein function can be assessed using the SIFT prediction incorporated in the database. Since SIFT predicts the extent of the mutation’s deleterious effect, the influence of stronger alleles on a phenotype/response can be compared in tandem with that of weaker alleles. While SIFT predicts the loss-of-function mutations, unfortunately, there are no similar tools for predicting the gain-of-function mutations.

Though genic mutations are the main contributors to phenotypes, emerging evidence indicates that promoter, UTRs, and intronic mutations also affect phenotypes by influencing gene expression. Likewise, mutations in miRNA genes affect the post-transcriptional regulation of several genes. The synonymous mutations are worth examining, as these mutations often influence a trait due to the organismal bias for codon usage. It is reported that synonymous mutations constitute approximately one-third of CDS mutations (Thompson et al., 2013; Henry et al., 2014). Our analysis is also consistent with this, as 37.39% of mutations in CDS were synonymous.

Unlike SIFT, the influence of the above mutations cannot be *a priori* predicted; the confirmation of their mutagenicity requires a detailed phenotype/biochemical examination. Nonetheless, the WGS is superior to the WES, as it reveals variants that WES does not discover. The WES largely excludes intronic variants and promoter mutations, as the emphasis is on the CDS region. It is increasingly becoming evident that the alteration of phenotypes is not restricted to genic variants, but intergenic region variants also contribute much.

### WGS provides a broader resource for trait improvement

Functional genomic analysis of non-coding mutations has emphasized their role in key biological processes in plants (Li et al., 2017). In tomatoes, the non-coding RNAs, circular RNAs, and miRNAs regulate protein coding gene expression through diverse mechanisms (Ma et al., 2020; Zuo et al., 2020). Our WGS analysis revealed a significant number of mutations in non-coding regulatory elements encompassing promoters, introns, and 5′- and 3′-UTRs. Our resource includes mutations in miRNA, and several of these reportedly regulate development in tomatoes (Ma et al., 2020; Zuo et al., 2020). The availability of non-coding mutations expands the mutation spectrum to discover elements regulating gene function or a biological pathway in tomatoes.

One may argue that owing to high background mutations, the assessments of gene function may be difficult. However, for genetic mutations, the SIFT predictions are quite reliable and can be used as a starting point. Additionally, examining multiple alleles of a given gene generally overshadows the effect of the background mutations. The mutations can be first validated by Sanger sequencing. To confirm genotype and phenotype cosegregation, the Mutmap approach can be used (Garcia et al., 2016), followed by backcrossing.

After backcrossing, the resulting seeds will be germinated and at the seedling stage, the mutated gene can be identified in the heterozygous and homozygous state using CEL-I, a mismatch-specific endonuclease. The heterozygous plants carrying the mutation can be recurrently backcrossed with the desired cultivar (Sharma et al., 2021; **Figure S15**). With the mutated gene itself being a marker, in the BC_4_F_1_ generation, 98% of mutations are eliminated (Hospital, 2003). In BC_4_F_2_, the homozygous mutant plants can be subjected to WGS to select the nearest isogenic BC_4_F_2_ line to the foreground cultivar.

### Induced mutagenesis by EMS vis-à-vis genome editing

Recently, the CRISPR/Cas9-based genome-wide editing for CDS has been applied to rice (Lu et al., 2017; Meng et al., 2017) and soybean (Bai et al., 2020). The genome-edited mutagenesis requires plant transformation with a large number of gRNA constructs designed *a priori* to disrupt the function of selected genes. The constructs are individually made and multiplexed-pooled to ease the large-scale transformation. The genome-edited plants are identified using standard protocols for identifying transgenic plants and validating editing.

In rice (Lu et al., 2017; Meng et al., 2017) and soybean (Bai et al., 2020), mutagenesis by genome editing mainly generated deletions. A more rigorous analysis in maize revealed that most edited genes had deletion (60%) than insertion (32.5%). In the remaining 8% of genes, two-thirds had transversions, and one-third had transitions (Liu et al., 2020). Broadly, genome-edited mutagenesis is similar to fast-neutron mutagenesis, which largely generates insertions and deletions (Li et al., 2017). In contrast, the EMS-induced mutations are mainly transitions, while transversions, InDels, and insertions are less frequent. The spectrum of mutations generated by EMS and genome editing is widely different. While genome editing generates mainly null or amorphic mutants, EMS mutagenesis provides a broader range encompassing amorphic, hypomorphic, hypermorphic, and antimorphic mutants. The CRISPR/Cas9-based mutagenesis essentially extends the repertoire of mutations in crop species by providing additional variants not generated by EMS. Notwithstanding the above, our fully sequenced mutant collections allow gene function investigation without the rigmarole of transformation and resources needed for genome editing.

## Conclusion

The WGS of mutagenized individuals uncovered the complete spectrum of mutations in the genome. We sequenced 132 doubly EMS-mutagenized lines of tomato and detected approximately 41 million SNPs and 5.5 million short InDels. Our gene-indexed genome-wide mutant repertoire provides a resource to the scientific community to functionally characterize a gene or a set of genes, including the unannotated genes. Our mutant resource will be helpful for studying a wide range of traits such as disease resistance, abiotic stress resistance, fruit ripening, and basic studies in functional genomics. Our data are available in the tomato genome database ITGV, where users can search for mutations in a desired gene or promoter and request the mutant seeds.

## Data availability statement

The data is available at University of Hyderabad at http://psd.uohyd.ac.in/itgv/.

## Author contributions

PG: Conceptualization, Data curation, Formal analysis, Methodology, Software, Writing – original draft. PD: Data curation, Formal analysis, Methodology, Software. KP: Investigation. AM: Investigation. YS: Funding acquisition, Project administration, Supervision, Writing – review & editing. RS: Conceptualization, Formal analysis, Funding acquisition, Project administration, Supervision, Writing – original draft, Writing – review & editing.
