# Supplementary material for: Augmenting tomato functional genomics with a genome-wide induced genetic variation resource
Source: Front Plant Sci. 2024 Jan 24;14:1290937. doi: 10.3389/fpls.2023.1290937 (PMC10848261; doi:10.3389/fpls.2023.1290937)
Supplement: Supplementary file 1 [file DataSheet_1.pdf]

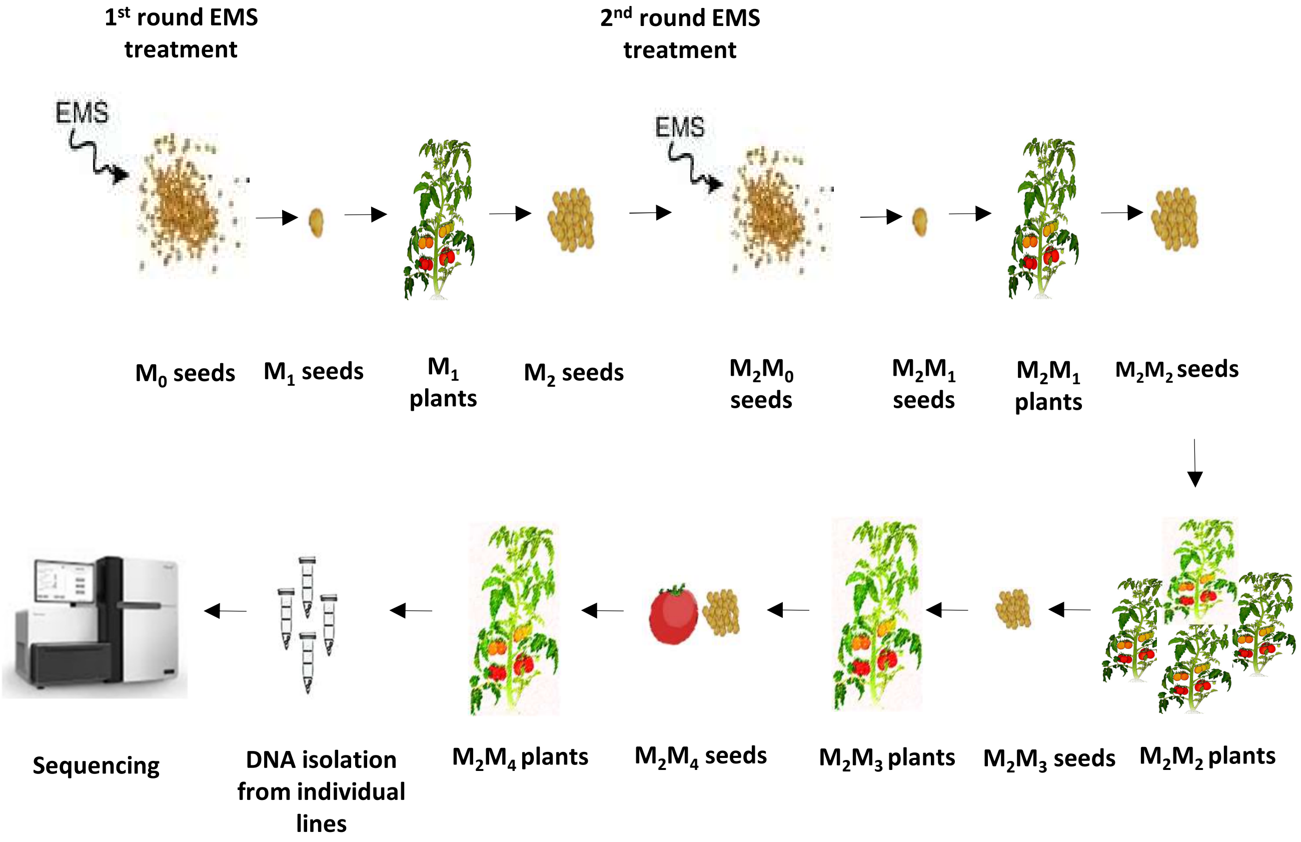

Figure S1. Schematic representation and structure of the EMS re-mutagenized population. The EMS-mutagenized (120 mM) Arka Vikas tomato cultivar (*Solanum lycopersicum*) M_2 seeds were remutagenized with 120 mM EMS. The remutagenized M_2M_1 seeds were used to raise M_2M_4 plants. The leaf tissue from 2-week-old M_2M_4 seedlings and the parental line Arka Vikas were harvested for genomic DNA isolation. The genomic DNA was subjected to WGS using the Illumina platform.

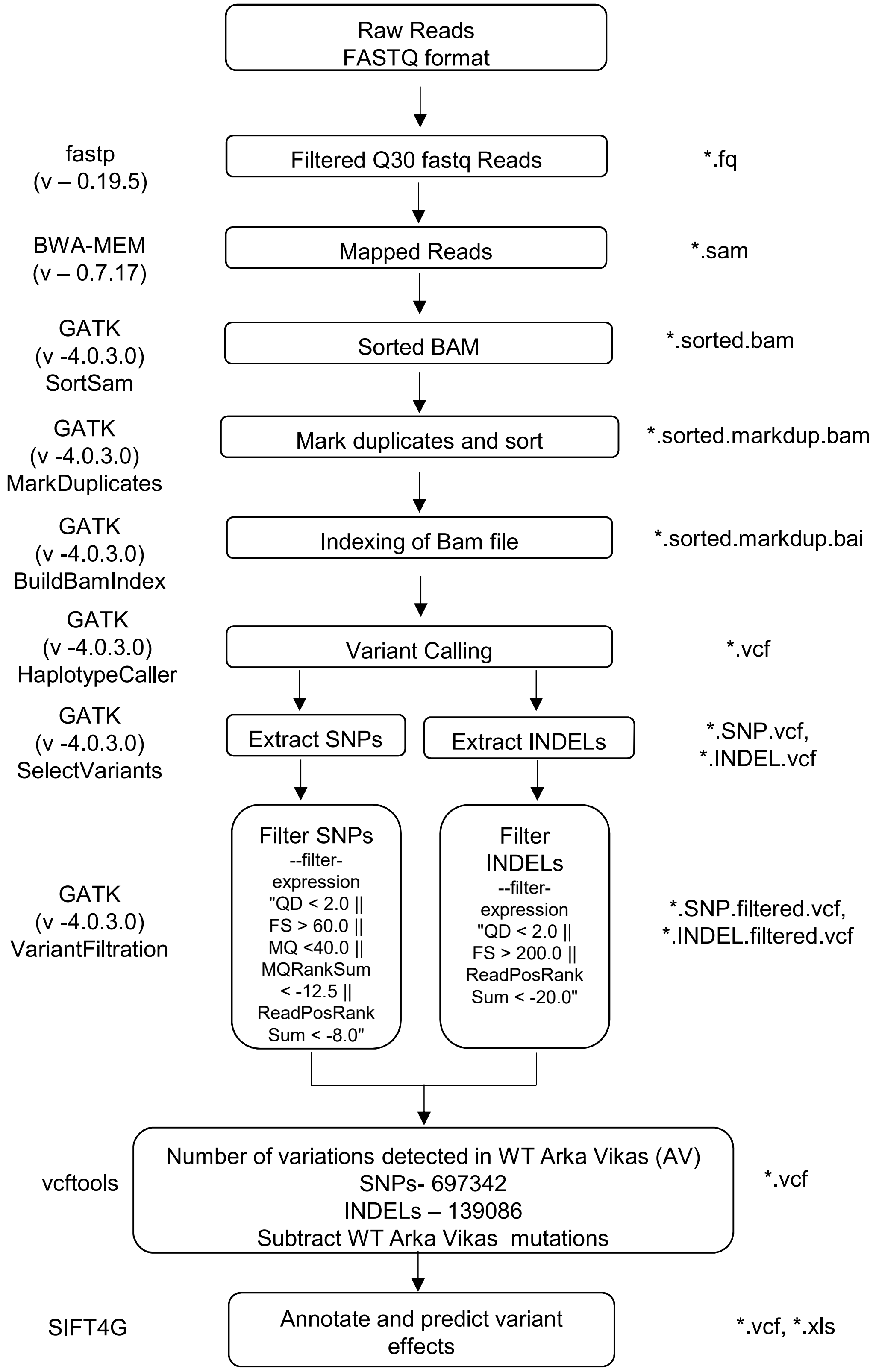

Figure S2. Variant calling pipeline used for calling SNPs and INDELs in EMS-mutagenized lines. The SNPs and INDELs present in the parental cultivar Arka Vikas were subtracted from all 132 mutant lines. The resulting vcf (variant calling format) files were annotated using the SIFT4G algorithm.

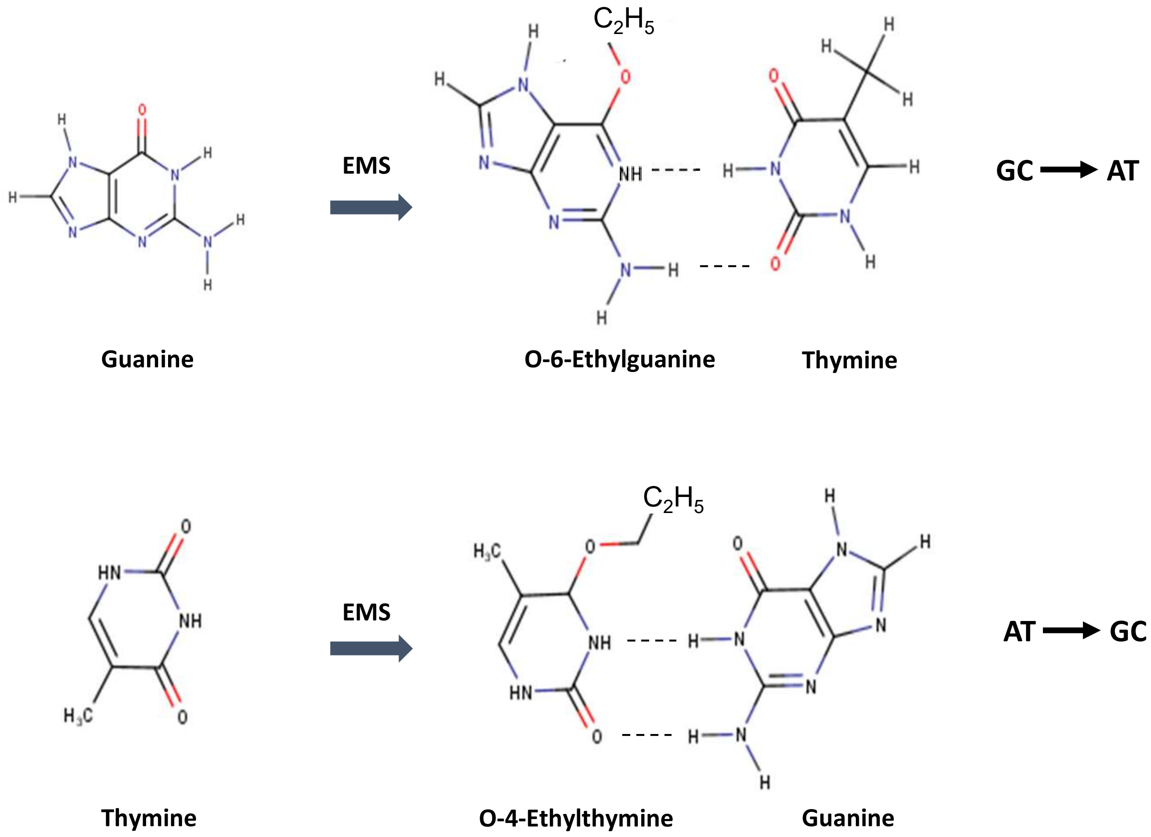

Figure S3. EMS-induced nucleotide mispairing leading to GC>AT and AT>GC transitions. EMS alkylates the O^6 position of guanine and the O^4 position of thymine, leading to mispairing with thymine and guanine, respectively.

.

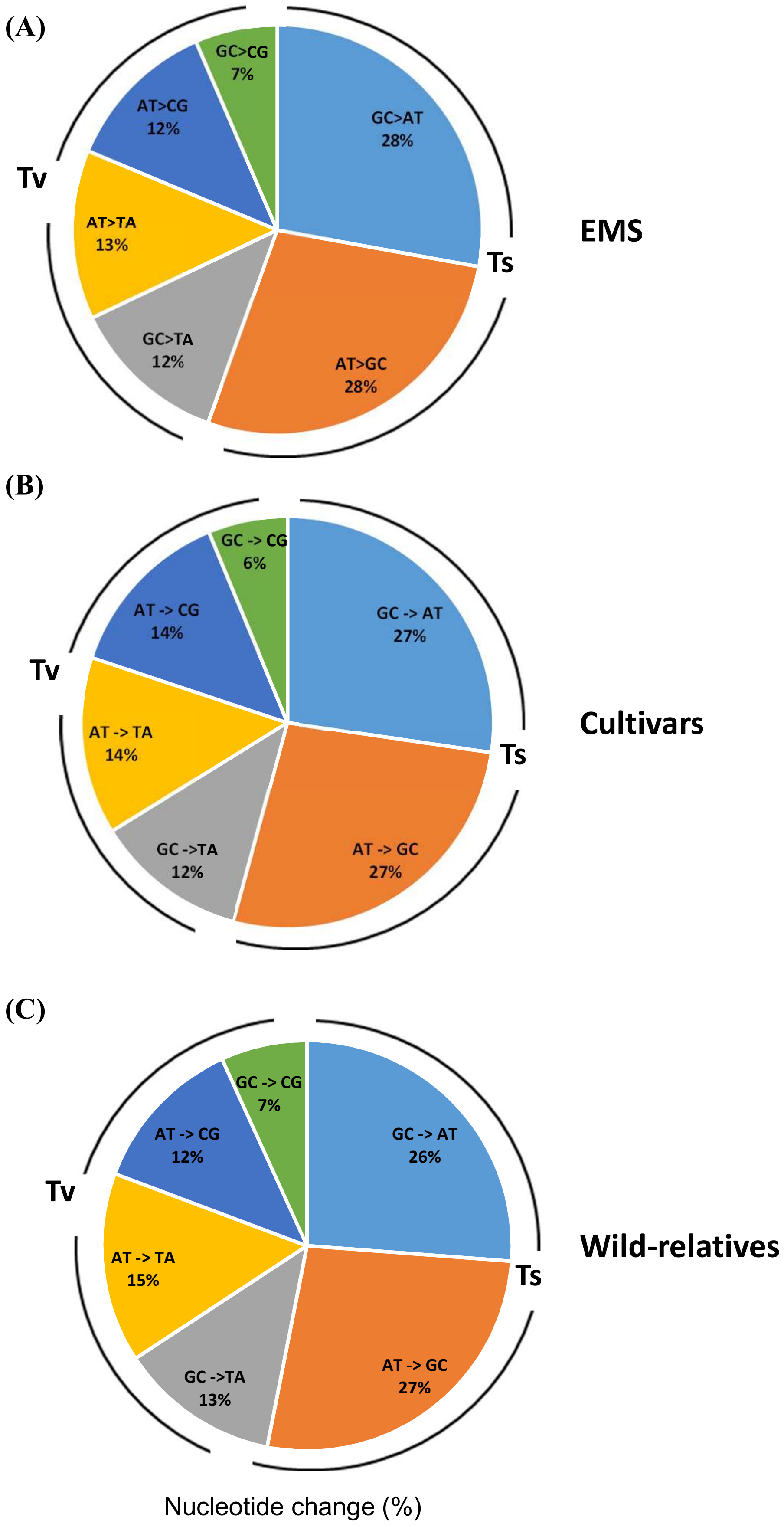

Figure S4. Pie charts showing the frequency of transitions and transversions. (**A**) EMS (Arka Vikas), (**B**) tomato cultivars, and (**C**) wild relatives. **Ts**- transitions, **Tv**- transversions.

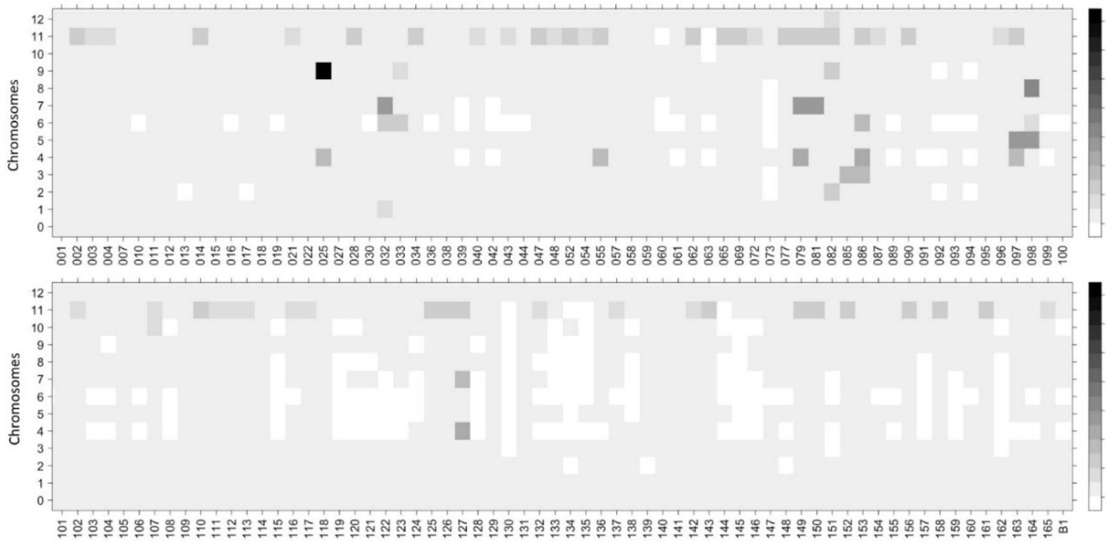

Figure S5. Distribution of the mutations across 12 chromosomes in the genome. Heatmap shows the distribution of the total number of SNPs in the 132 mutant lines.

(A)

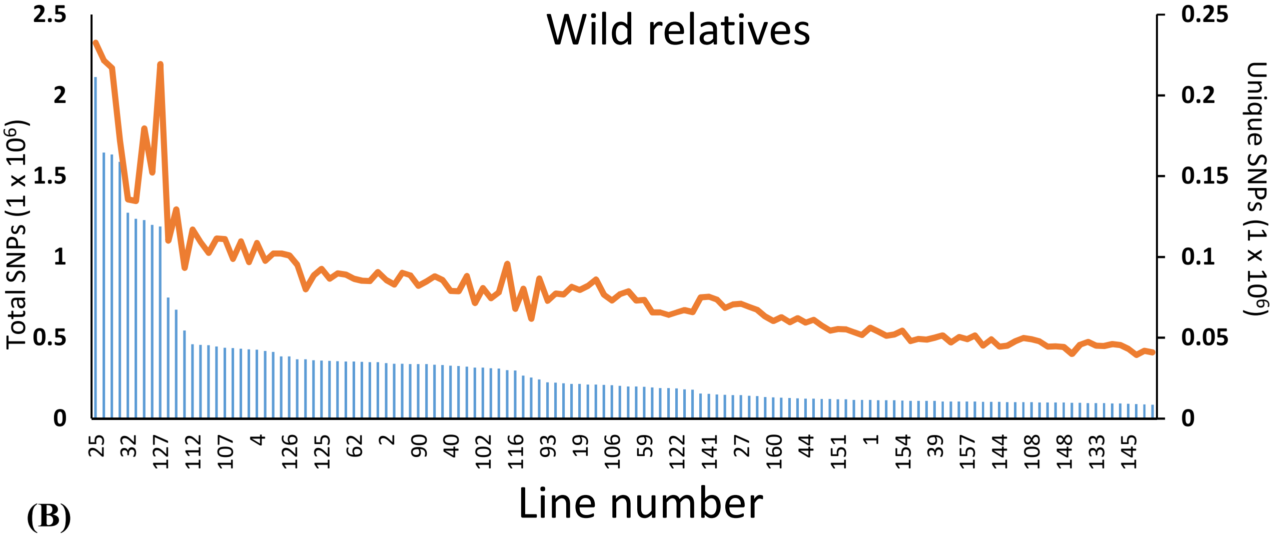

(B)

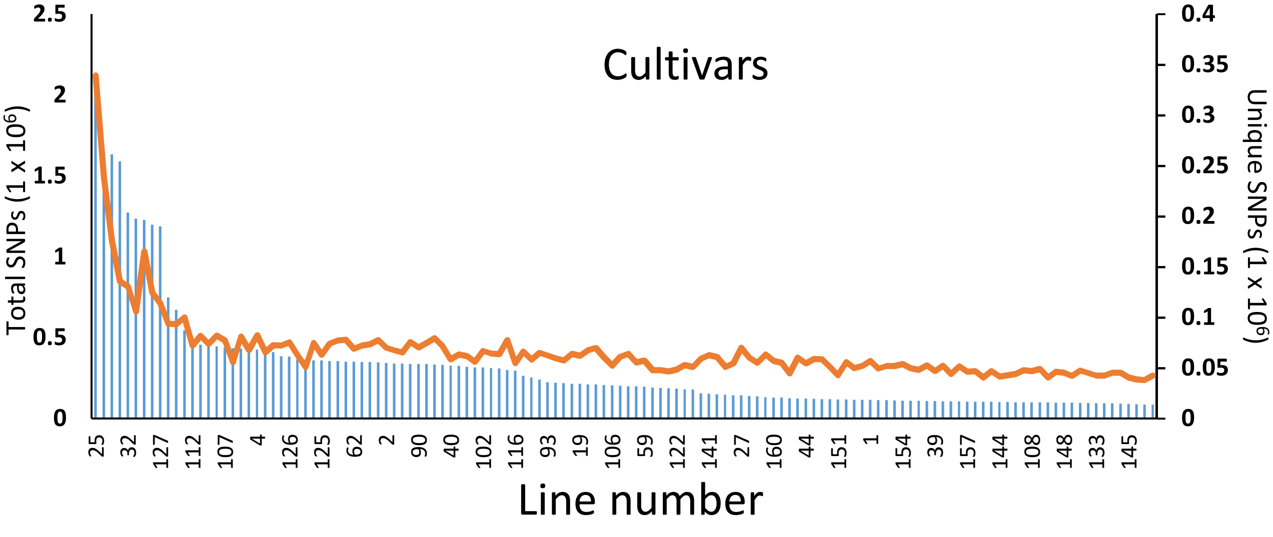

Figure S6. The total number of unique SNPs present in 132 mutant lines compared to 30 wild relatives of tomato (**A**) and 54 tomato cultivars (**B**). For details, see Table S6.

(A)

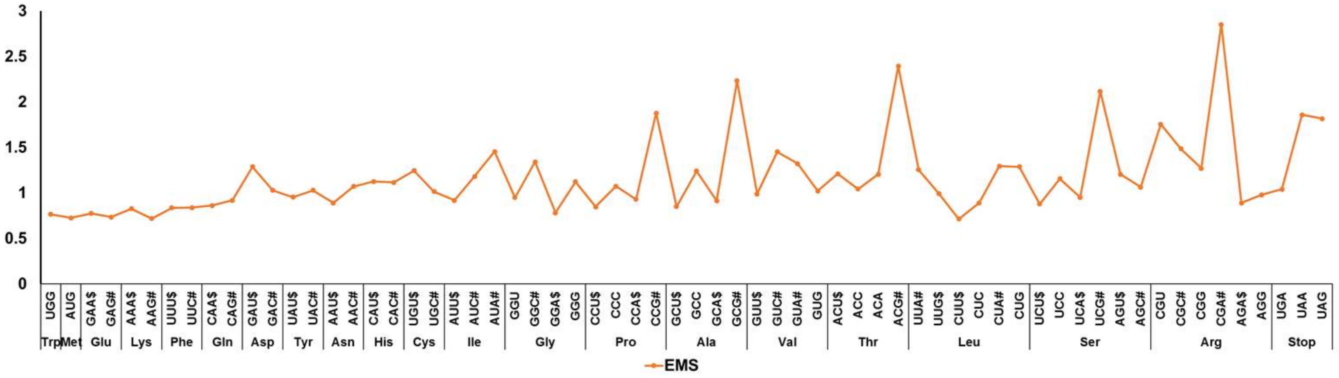

(B)

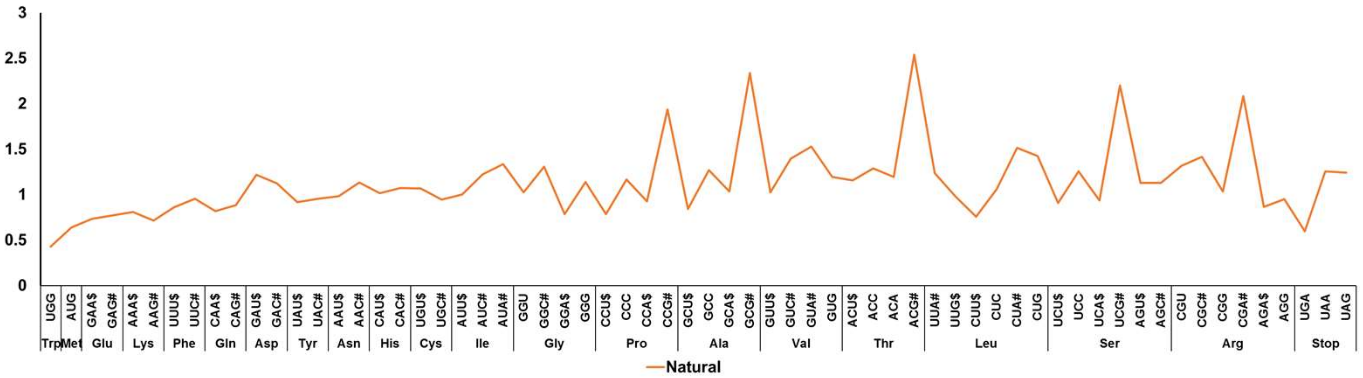

(C)

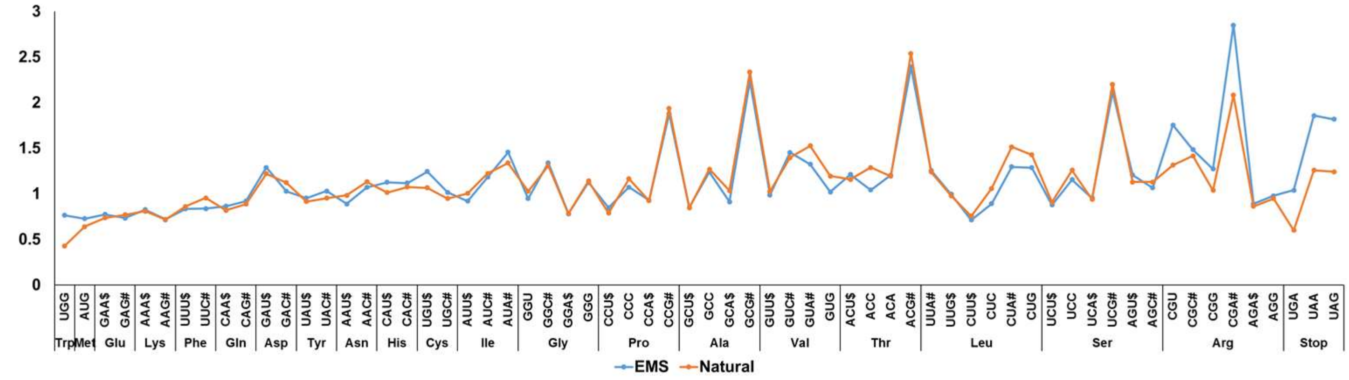

Figure S7. The ratio between the frequency of a mutated codon and its normal frequency in tomato. (**A**) EMS, (**B**) Natural and (**C**) EMS v/s Natural. The most preferred codon, as per the tomato codon usage table (https://solgenomics.net/documents/misc/codon_usage/codon_usage_data/l_esculentum_codon_usage_table.txt), is marked with the **$** dollar sign, and least preferred codon is marked as **#** hashtag after the codon letter in the graph. The individual ratios are given in Table S12.

Cellular component

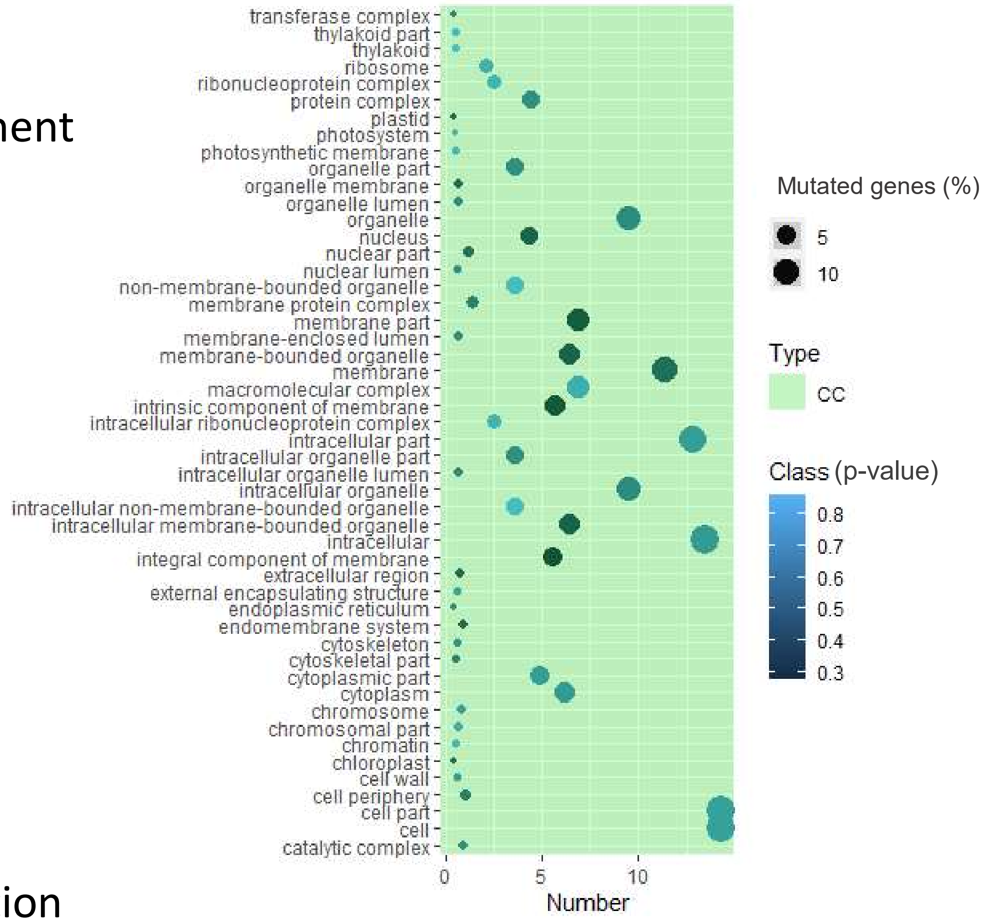

Molecular function

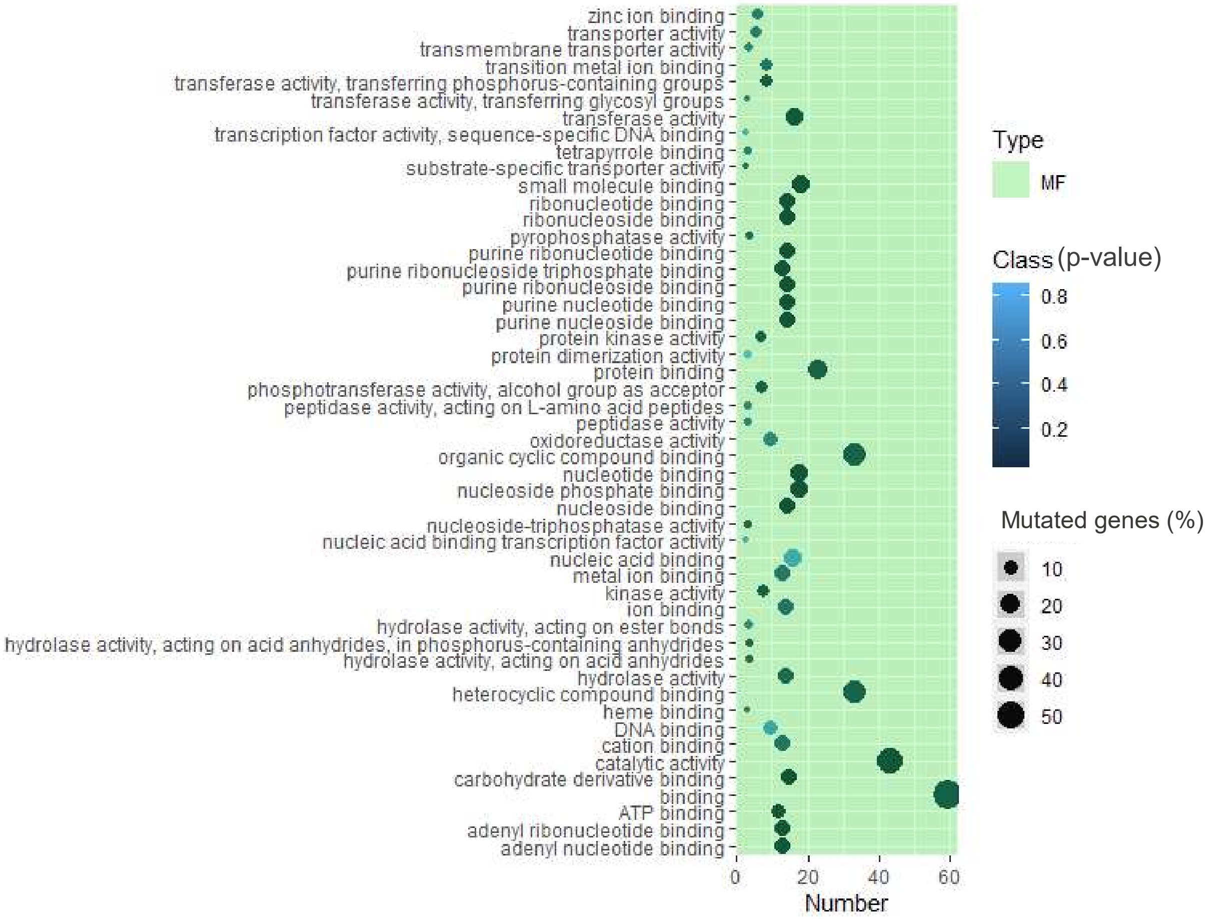

Figure S8. Distribution of mutations in Cellular component and Molecular function GO categories. Top 50 GO categories with the high frequency of mutations in Cellular component and Molecular function. For details, see Table S15.

(A)

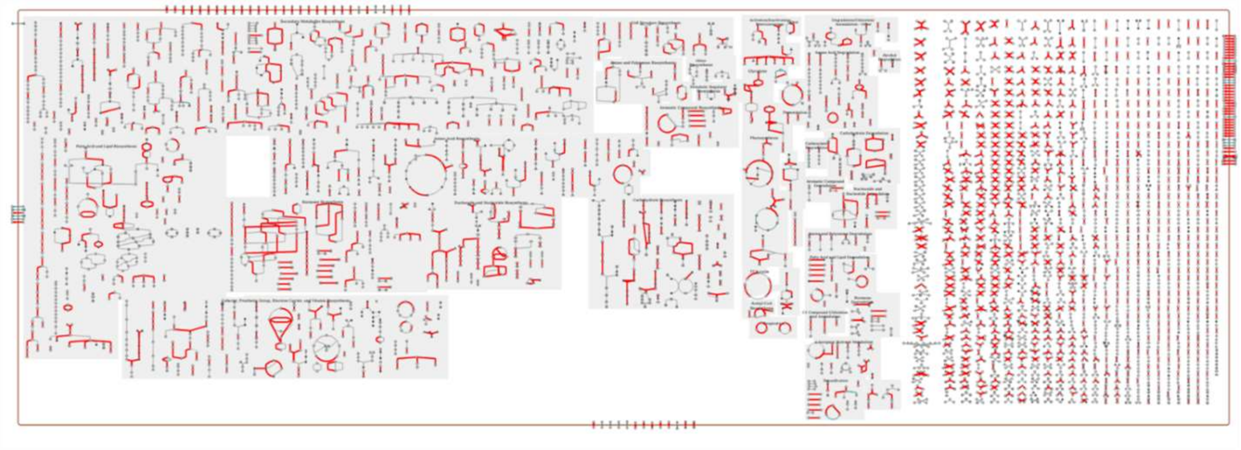

(B)

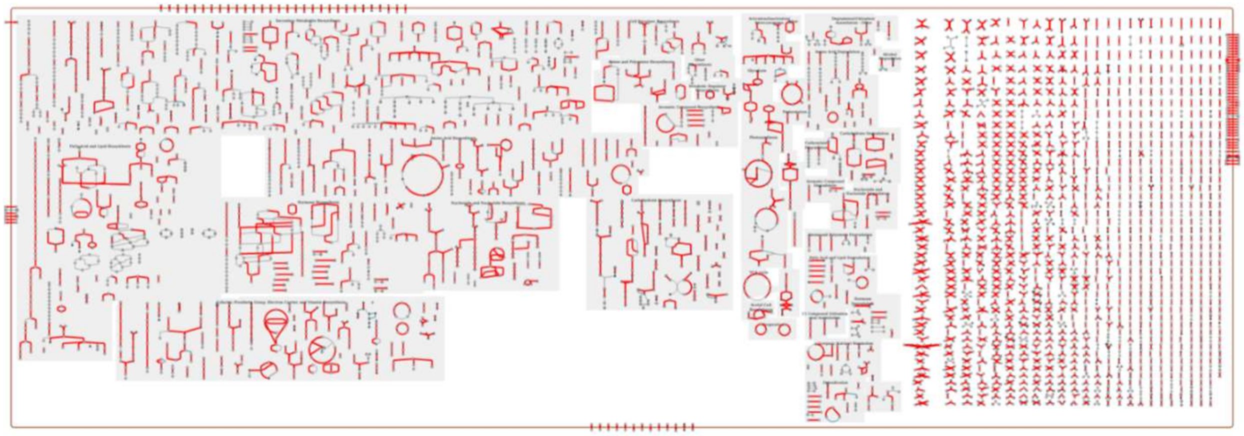

Figure S9. Distribution of mutations on different metabolic pathways. (**A**) Tomato metabolic pathway steps are marked with deleterious mutations present in the mutagenized population. For details, see Table S10. (**B**) Tomato metabolic pathway steps marked with non-synonymous mutations present in the mutagenized population. Gene Id's were mapped to the tomato metabolic network from Plant Metabolic Network Database (https://pmn.plantcyc.org/overviewsWeb/celOv.shtml?orgid=TOMATO).

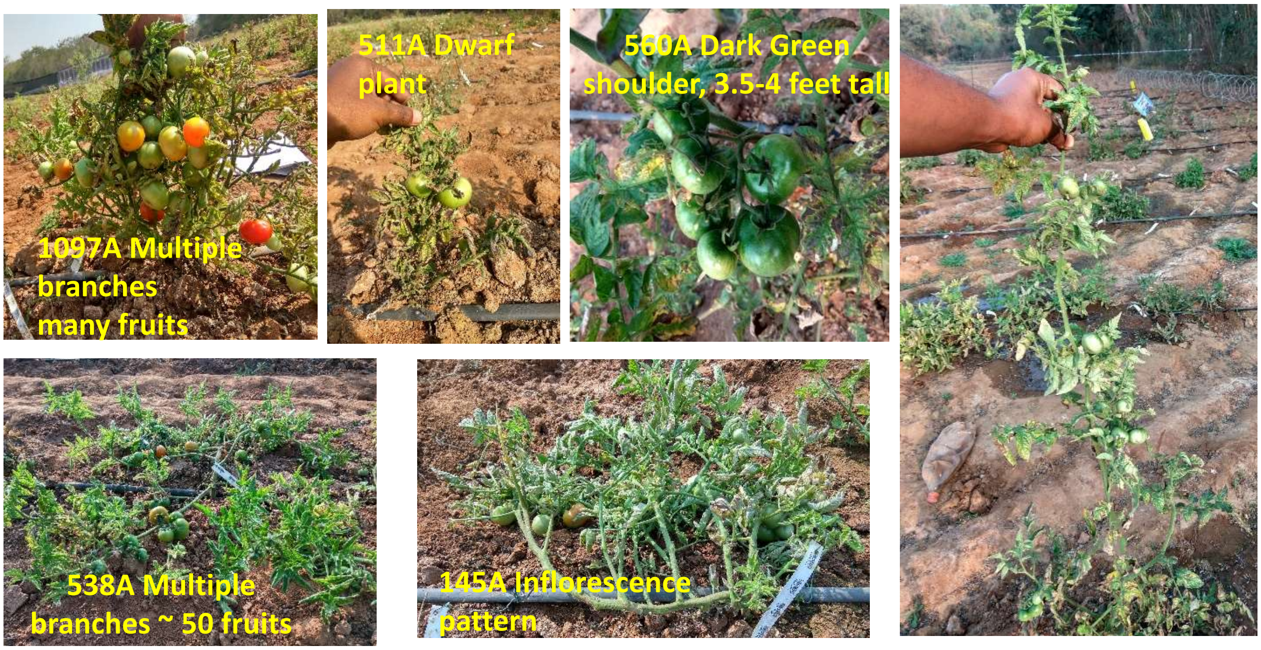

Figure S10: Morphological diversity of various mutant lines showing aberrant developmental phenotypes.

.

(A)

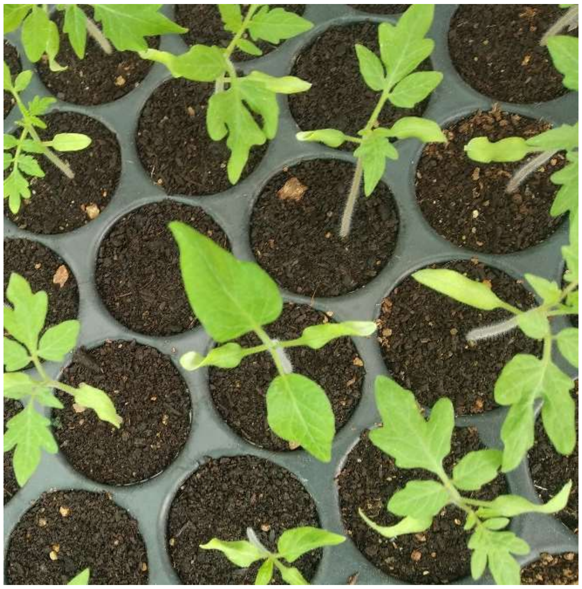

(B)

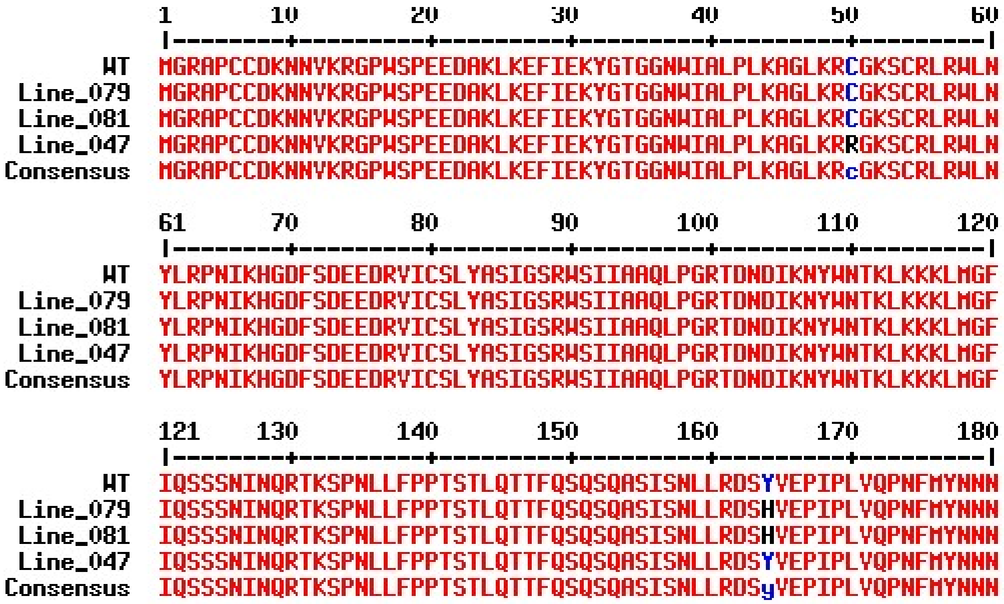

Figure S11: RM277A line with leaves displaying the potato leaf phenotype (**A**) RM277A (Line no. 047) line with leaves displaying the potato leaf phenotype. The point mutation was validated in the MYB transcription factor (Solyc06g074910) encoding the potato leaf locus (Locus c). (**B**) Protein sequence alignment of Lines 047, 081, and 079 showing amino acid changes.

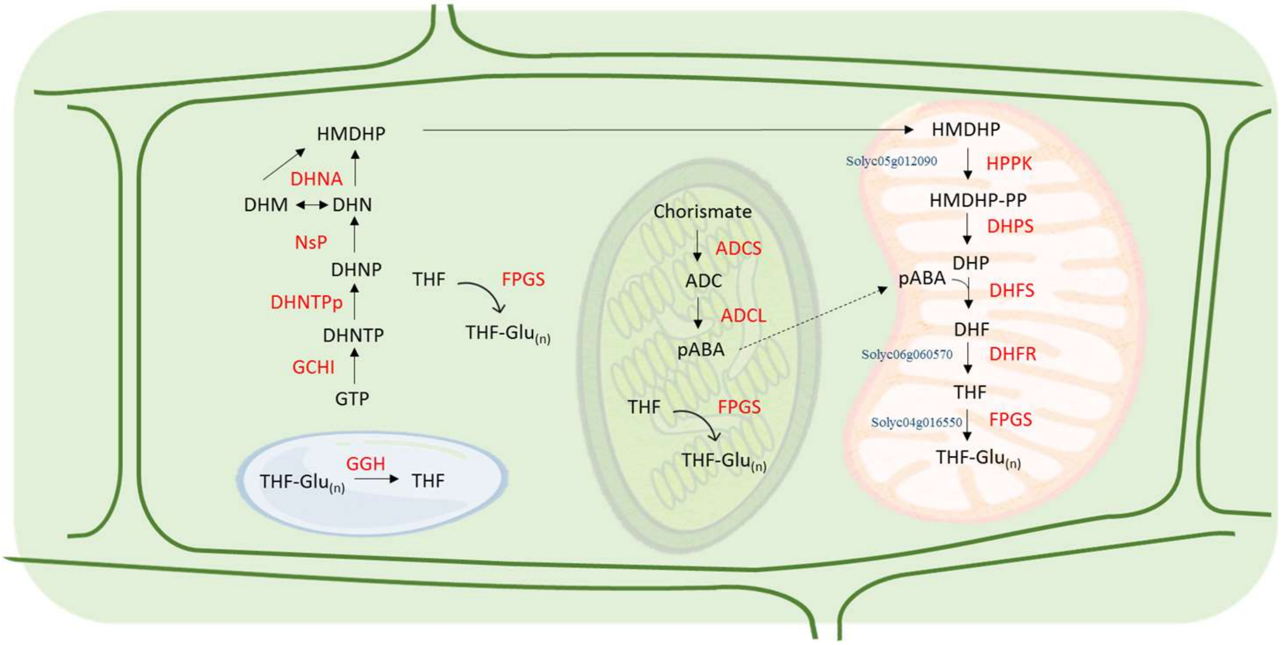

Figure S12. Folate biosynthesis pathway marked with deleterious mutations present in the mutagenized population. The gene id's are marked in blue on different steps of the pathway. Note that the folate biosynthetic pathway is recalcitrant to mutagenesis, as folate is essential for nucleotide synthesis and C1 metabolism. For details, see Table S15.

Abbreviations: *Precursors:* GTP, guanosine triphosphate; DHNTP, dihydroneopterin triphosphate; DHNP, dihydroneopterin monophosphate; DHN, dihydroneopterin; HMDHP, 6-hydroxymethyldihydropterin; HMDHP-PP, 6-hydroxymethyldihydropterin pyrophosphate; DHP, dihydropteroate; DHF, dihydrofolate; THF, tetrahydrofolate; THF-Glu$_{(n)}$, tetrahydrofolate polyglutamate; ADC, aminodeoxychorismate; pABA, para-aminobenzoic acid. *Enzymes:* GCHI, GTP cyclohydrolase I; DHNTP$_P$-diphosphatase, dihydroneopterin triphosphate pyrophosphatase; DHNA, dihydroneopterine aldolase; HPPK, HMDHP pyrophosphokinase; DHPS, dihydropteroate synthase; DHFR, dihydrofolate reductase; FPGS, folylpolyglutamate synthetase; ADCS, aminodeoxychorismate synthase; ADCL, aminodeoxychorismate lyase; GGH, gamma-glutamyl hydrolase.

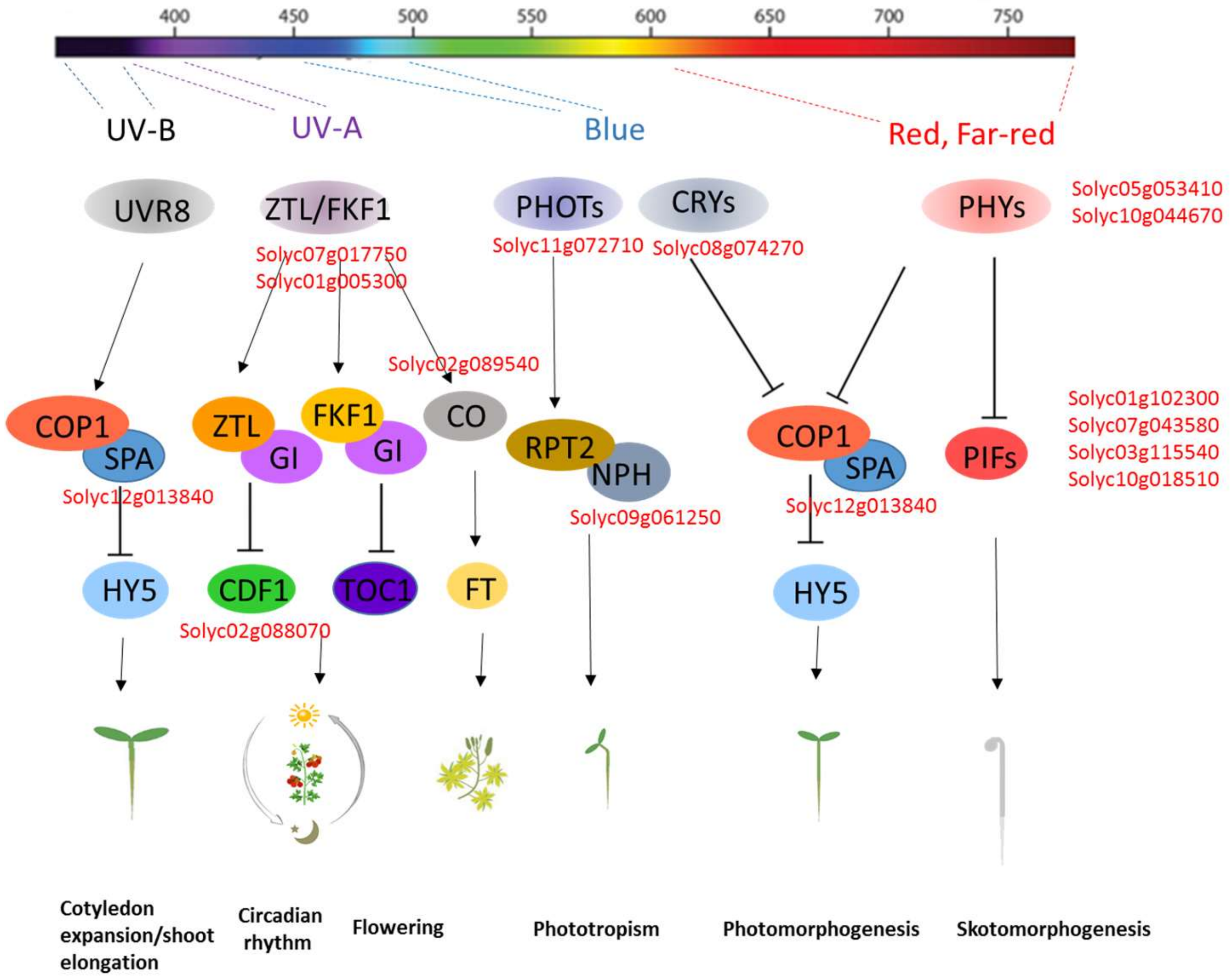

Figure S13. Light-signaling pathway marked with deleterious mutations present in the mutagenized population. The gene id's are marked in red on different steps of the pathway.

Abbreviations: UVR8, UV-B resistance 8; COP1, Constitutive Photomorphogenic 1; SPA, Sugar partitioning Affecting protein; HY5, Elongated Hypocotyl5; ZTL, ZEITLUPE; FKF1, Flavin-binding, Kelch Repeat, F-BOX 1; GI, GIGANTEA; CDF1, Cycling Dof Factor 1; TOC1, Timing Of Cab Expression 1; CO, CONSTANS; FT, Flowering Locus T; PHOTs, Phototropins; RPT2, Root Phototropism protein 2; NPH, Non-Phototropic Hypocotyl; CRYs, Cryptochromes; PHYs, Phytochromes; PIFs, Phytochrome interacting factors.

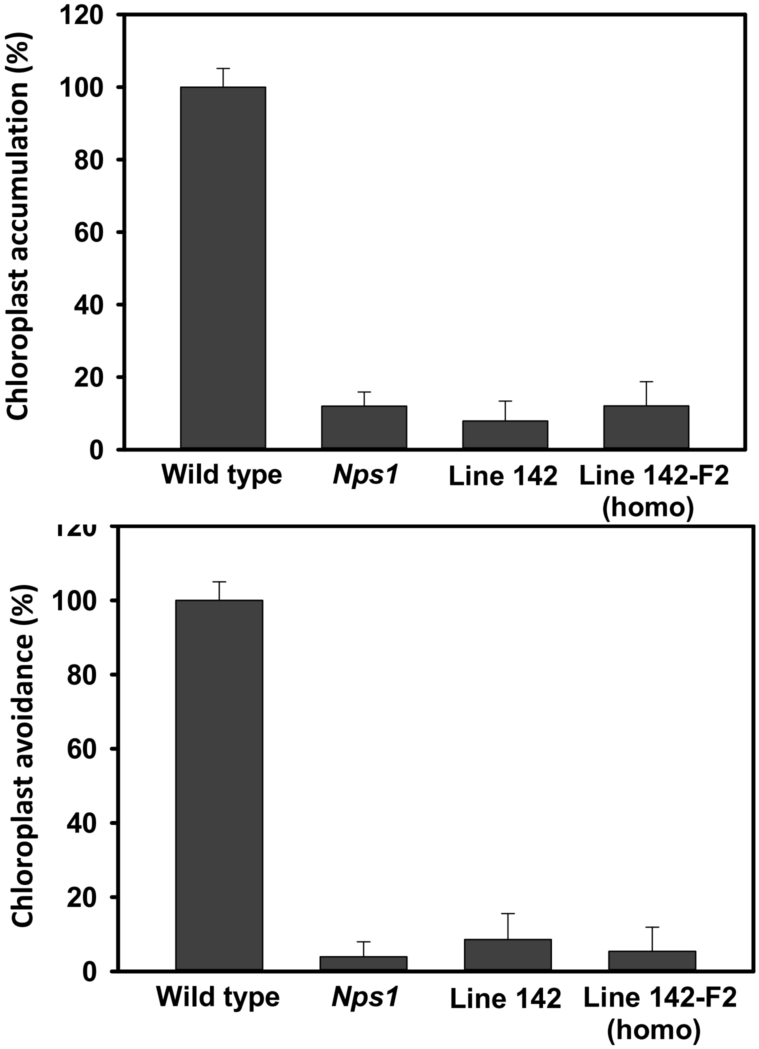

Figure S14. The chloroplast relocation response in leaves of mutant line 142 and its backcrossed progeny. Like the *Nps1* mutant, both lines show near-total loss of chloroplast relocation response in the parental line as well as in the F_2 generation.

.

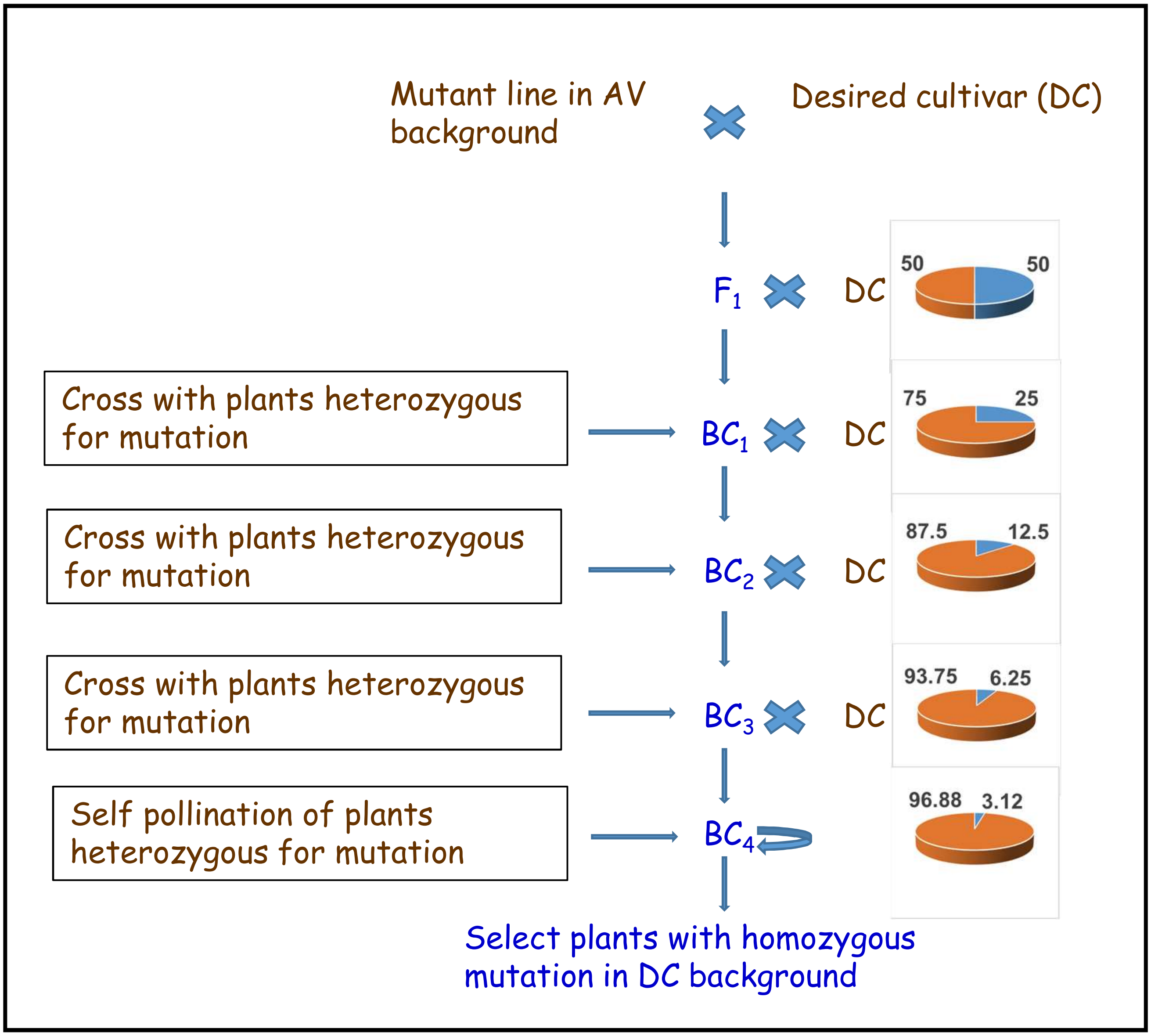

Figure S15: The theoretically expected recovery of the recurrent parental line background on backcrossing (red pie). Since the mutated gene itself acts as a marker, the expected recovery by BC_4 is ≥ 99% of the recurrent parental genome (**Hospital F**. 2003 Marker-assisted breeding**. In:** H.J. Newbury, editor. *Plant Molecular Breeding*. Oxford and Boca Raton: Blackwell Publishing and CRC Press; p. 30-59). By doing WGS of the BC_4F_2 lines homozygous for mutations, the near-isogenic lines to the recurrent parent can be recovered.
